# Agent-based simulation of trust networks and opportunistic behaviours of hydraulic infrastructure project participants

**DOI:** 10.1371/journal.pone.0316992

**Published:** 2025-01-06

**Authors:** Jiaqi Lv, Xiang Wang, Yaoyao Chen

**Affiliations:** 1 College of Statistics and Data Science, Lanzhou University of Finance and Economics, Lanzhou, Gansu, P. R. China; 2 School of Management Engineering, Qingdao University of Technology, Qingdao, Shandong, P. R. China; 3 College of Engineering and Physical Sciences, University of Birmingham, Birmingham, West Midlands, United Kingdom; University of Klagenfurt, AUSTRIA

## Abstract

Opportunistic behaviour has become a research hotspot in hydraulic infrastructure project management owing to its serious damage to the cooperation efficiency of all participants in a project. The trust networks formed by each participant can restrain opportunistic behaviour, but due to the dynamic evolution of the networks, its research should adopt a dynamic paradigm. The structure and evolution of trust networks can be simulated using computer simulations and modelling. This study explores the influence mechanism of the structural characteristics of trust networks on the diffusion of opportunistic behaviour. The results show that trust network density has a positive effect on inhibiting the implementation tendency of opportunistic behaviour. However, the centralisation of trust networks has a negative correlation with the degree of inhibition of opportunistic behaviour.

## Introduction

The successful completion of a hydraulic infrastructure project requires the cooperation of all participants to fulfill the engineering task and realize the project’s value. In fact, compared with conventional construction projects, hydraulic infrastructure projects require more different types of participants to fulfill their duties [1]. Whether it is a dam, dike or conservancy pipeline project, its design and use standards, the amount of concrete pouring, the number of hidden works are more complex than conventional construction projects [2]. What is more serious is that if the hydraulic infrastructure project brings quality risk due to the opportunistic behavior of the participants, the damage caused by it will be extremely serious, which is incomparable to conventional construction projects. Therefore, it is very important to optimize the relationship quality of participants in hydraulic infrastructure projects, improve cooperation efficiency, and suppress opportunistic behavior.

However, previous studies on the relationship governance of hydraulic infrastructure projects mainly take a binary perspective, that is, the analysis is conducted around the relationship between the employer and the contractor [3]. But hydraulic infrastructure projects are indeed more than its composing dyads, and looking at it as a sum of dyadic relationships does not allow managers and researchers to take into account the complex web of interdependencies which characterizes the project participants and influences behaviours and performance outcomes in real-world project [4]. At the same time, hydraulic infrastructure projects have significant differences from traditional engineering projects. hydraulic projects require a large number of participants to work together over a long period of time, so that from the site selection, design, construction and operation stages, all parties need to participate in addressing various risks and challenges, rather than independently conducting closed work. Thus, doing impactful hydraulic infrastructure projects management research may benefit from shifting the level of analysis from the dyad to the overall network. Based on the above, trust has always been considered as a core factor in the relationship governance mechanism of hydraulic infrastructure projects. However, most studies on trust in hydraulic infrastructure projects contexts have focused on dyadic relationships. Conversely, there has been a paucity of research on trust at the level of the overall participants network [5].

In addition to contractual and administrative constraints, each participant in the project must also adhere to the internal management system. These institutional arrangements are typically outlined in specific forms such as internal management manuals or joint-work programs, primarily developed and implemented by the project owner. The internal management system significantly influences the behavior and engineering tasks of each participant, including design scheme adjustments, quality management, milestone payments, and collaborative work. Governance measures related to trust networks will impact all participants within the internal management system of the project [3]. For this study’s purposes, trust networks (i.e., network-level trust) represent how pervasive trust is among hydraulic infrastructure project participants [6]. Social network theory emphasizes that network structure can influence individual behaviors within a network and subsequently affect overall performance [7]. However, it is not clear how trust network structure influences the specific behavior of each project participant, which is the main research objective of this study. The conclusions drawn from this study will provide insights into diverse internal management systems within hydraulic infrastructure projects.

## Theoretical background

### Trust networks of project participants

Trust relationships are an effective way ensuring the efficiency of cooperation and improving the performance of construction projects to restrain the opportunistic behaviours of all participants [8]. In this regard, previous research has primarily focused on the analysis of the owner and contractor of engineering projects, but ignored the complex social network relationships embedded in these projects [3]. Social network theory emphasises that both economic and sociological phenomena should be explained from the perspective of networking; that is, the characteristics of network structure have a profound impact on the overall performance by influencing the specific behaviours of each participant, which is also the main research paradigm of Social Network Analysis [5]. Previous studies have pointed out that trust relationship plays a unique role in knowledge transfer in the context of social networks, which undoubtedly provides favorable evidence for the impact of trust relationship on organizational performance [9]. Similarly, previous studies based on transaction cost economics show that the trust relationship between participants plays an important role in inhibiting opportunistic behaviours, but the binary relationship between project employer and contractor is still taken as the main research perspective [10]. The good news is that previous studies have used case analysis and empirical research methods to explore the following aspect: network-level trust to restrain opportunistic behaviour and enhance cooperation efficiency of all stakeholders [4]. On this basis, previous research in the supply chain or enterprise management also has discussed the network structure of trust relationships, and mainly explained the influence of network-level trust on the specific behaviour or performance of each participant through case studies [11].

However, empirical studies using cross-sectional data tend to overlook the frequent and complex dynamic interactions between project participants, while case studies often lack a broad representation and rigorous analysis processes [12]. It is undeniable that the completion of construction projects requires the joint efforts of many participants, so there is objectively a relationship of trust or distrust between them, and then constitute a complex network structure [13]. In fact, when the perspective is expanded from binary trust between two subjects to diversified trust networks, the influence path and interference degree of this network structure on the opportunistic behaviour and cooperation efficiency of each participant in the construction project is not clear [6].

### Influence of trust networks on behaviour

Social network theory emphasises that the nodes embedded in the networks are affected by other nodes and the overall structure of the network, so the behaviour choice and result direction of a node are not only determined by the node itself, but also regulated by the network structure embedded in it [7]. Based on this theory, previous studies have shown that behaviours of participants can be contagious or suppressed through social networks [14]. In this regard, from the perspective of public-private partnership (PPP) projects, existing studies have analysed that the structural characteristics of trust networks have heterogeneous correlation with the opportunistic behaviour of each participant, that is, the network density is negatively correlated with the opportunistic behaviour, and the centralization index is positively correlated with the behavior [6]. Similarly, previous studies used the social network analysis (SNA) to characterize the trust networks structure between stakeholders of PPP projects, and analysed the possible impact of structural holes or faction alliances on opportunistic behaviours [15]. However, it is still not clear whether the transmission or suppression of opportunistic behaviours of various participants in construction projects is influenced by the network-level trust relationship [15]. That is, from the perspective of social network theory, whether the actualization tendency and propagation of each participant’s opportunistic behaviour can be influenced by the network structure of trust relationship.

**Fig 1:**
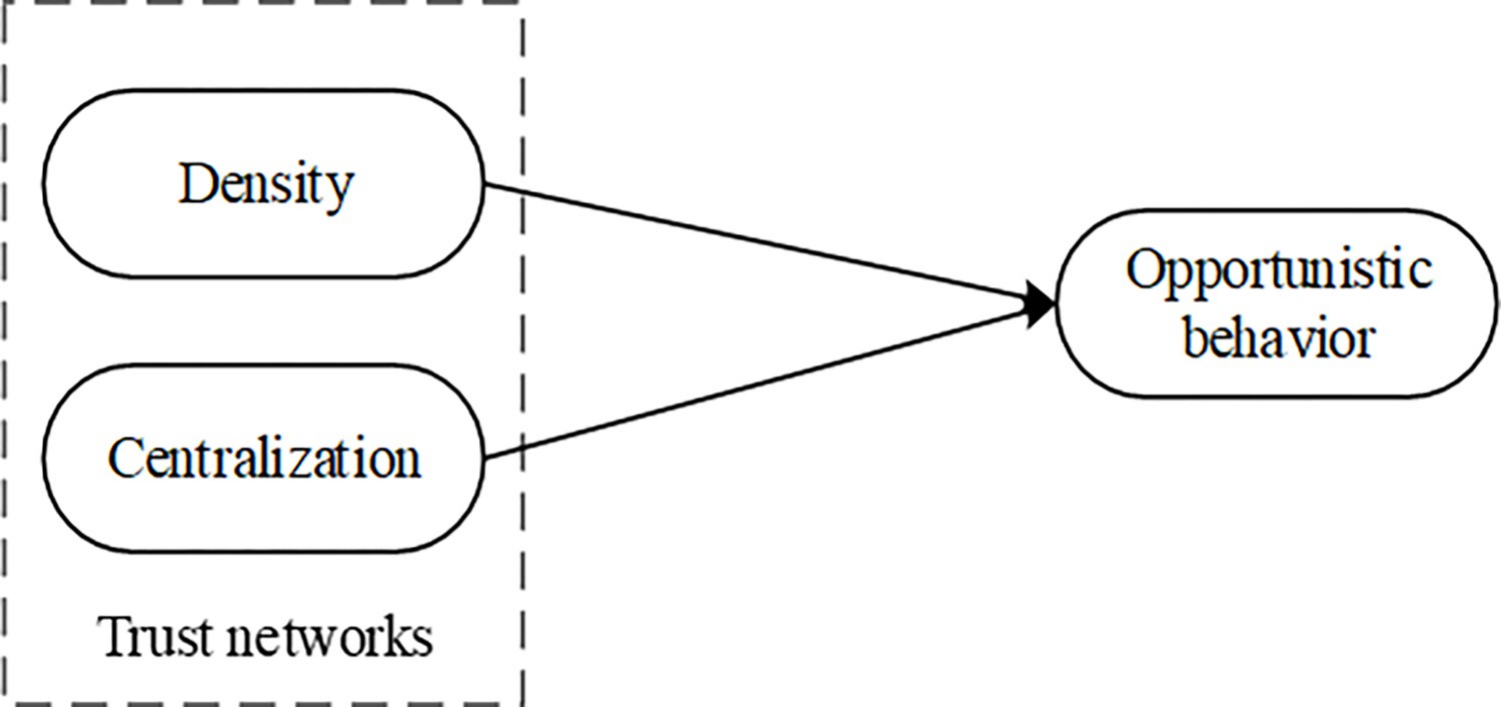
Conceptual model.

In fact, previous studies have pointed out that different characteristics of social network structure will have heterogeneous influence on the behaviours of various participants in the networks [16]. In this respect, the density and centralization of social network are the main indicators that can show the overall characteristics of network structure [17]. Therefore, the differences of different structural characteristics should be distinguished in the process of analysing the influence of trust networks on opportunistic behaviour and the conceptual model is shown in Fig 1.

#### Density and opportunistic behavior

Based on social network theory, network density can be denoted as the ratio of existing connections between nodes and the maximum number of possible connections in a social network [5]. Previous research has shown that the existence of trust connections between nodes is the main factor affecting the transmission of opportunistic behaviour [16]. On the contrary, it is also common in construction projects that there is no trust relationship between the project participants. In this case, the implementation tendency of the opportunistic behaviour of the project participants will not be affected by the trust relationship [18]. Furthermore, if there is a trust relationship between any two nodes in the networks, the node with higher opportunistic behaviour tendency will be influenced by other nodes and inhibit the implementation motivation of its own behaviour [19]. Specifically, the value of the trust network density is mainly affected by the number of connections and nodes. When the number of network nodes is relatively stable, we can assume that the density of the trust network negatively affects the opportunistic behaviour tendency of each node in the network [7].

#### Centralisation and opportunistic behavior

The centralisation emphasised by social network theory mainly reflects the degree of over-concentration or imbalanced connections between network nodes. According to the basic view of SNA, higher centralisation indicates that some nodes in the network have more connections, while simultaneously some have only a few or no connections [20]. Previous research based on social network theory has emphasised the influence of centralisation on the behaviour of nodes in networks [21]. For example, for the transformation of scientific and technological achievements in social networks, network centrality is negatively correlated, and network density is positively correlated with it. This indicates that the unequal status of each participant in the social network, or the farther the distance between each other, is not conducive to the implementation of positive behaviour.

Similarly, research on the knowledge-sharing behaviour of enterprise employees based on complex social networks found that the higher degree centralisation of social network structure indicates that the knowledge sharing behaviour right of each node in the network is excessively concentrated, which means that the knowledge sharing behaviour within the social network depends on a few nodes [19]. Therefore, a higher centralisation index can inhibit normal behaviour among nodes. Additionally, in the trust networks constructed by each participant in construction projects, the high centralisation level indicates that all participants trusted key nodes, while there were isolated nodes or nodes with only a few trust relationships. Through empirical analysis, previous research has shown that the centralisation of trust networks is positively correlated with opportunistic behaviour [6]. Therefore, it can be assumed that the centralisation of trust networks cannot suppress the spread and implementation tendency of opportunistic behaviour.

## Research methodology

Considering that hydraulic infrastructure projects have the basic characteristics of singleness and one-off, it is difficult to construct a network structure model that can represent multiple project types according to the information and data of a specific hydraulic infrastructure project [22]. Therefore, it is necessary to find an extensive and representative network structure model from the existing mature models and modify it according to the actual situation of hydraulic infrastructure projects to form a trust network model with universality as well as characteristics of hydraulic infrastructure projects [23]. In fact, the research method of simulation modelling can simulate the problems of time series and make the analysis results have good universality through the iteration of massive data, which effectively solves the problems exposed by case or empirical analysis methods in management or economics research [24]. Therefore, referring to the quantitative measurement and computer programming methods of immoral or non-ethical behaviour in previous research, we deconstruct and code the implementation tendency of opportunistic behaviour and interactive influence relationship of each participant in the hydraulic infrastructure project in the trust networks [25]. On this basis, we use agent-based modelling and simulation method to characterise the trust network structure of each participant in the hydraulic infrastructure project, and to discuss the influence of the implementation tendency of each participant’s opportunistic behaviour.

### Basic settings

Agent-based modelling and simulation (ABMS) was used to characterise the trust network structure of each participant in the hydraulic infrastructure project, and the NetLogo platform was mainly used for simulation and modelling operations [26]. Although the goal of simulation modelling is to simulate real and objective phenomena, there will be a slight difference between model hydraulic infrastructure and the real world due to the complex and changeable rules of opportunistic behaviour of project participants and the diverse factors affecting the opportunistic behaviour tendency of each participant. On that account, engineering practice is simplified in this study, which is embodied in the following modelling assumptions.

Each participant in the project is represented by a turtle on the NetLogo platform, the opportunistic behaviour tendency of each participant is the endogenous subjective will of the turtle, but it is affected by the trust network environment embedded by each participant in the project. The connection of trust relationships between any two participants will affect their opportunistic behaviour, which is consistent with the interaction between trust and opportunistic behaviour in previous studies [3]. The connections between the turtles represent the trust relationships between randomly linked builders.

Based on social network theory, there are several basic forms of network structure, such as regularization, randomization, small-world, and scale-free, while scale-free network is more suitable for describing the relationship between each participant in a hydraulic infrastructure project [27]. Moreover, the classical calculation formula of network density and centralisation in the social network analysis (SNA) method is used to represent the overall characteristic index of trust networks [28]. Concomitantly, considering the definition and interpretation of opportunistic behaviour tendencies in transaction cost theory, we can use the method of setting a random number for each turtle to indicate the initial tendency of each participant’s opportunistic behaviour [29]. As the simulation objective of this study is to explore the inhibition effect of the opportunistic behaviour tendency in trust networks by the trust relationship, individuals with high levels of opportunistic behaviour tendencies are affected by individuals with low levels of opportunistic behaviour tendencies.

### Behaviour rules

The implementation tendency of the opportunistic behaviour of each participant in a hydraulic infrastructure project is not completely clear, nor can it be accurately quantified. Therefore, the randomly set value of O_A1_ represents the initial opportunistic behaviour tendency of participant A, and O_A2_ represents the opportunistic behaviour tendency of participant A after the evolution of the trust network. Both O_A1_ and O_A2_ are within the range of [0,1], and the higher the value, the stronger the behaviour tendency. In the trust networks, the degree to which the implementation tendency of opportunistic behaviours of all participants is suppressed, is expressed by the change in behavioural tendency △O. For the specific measurement method, refer to Formula 1 in Table 1.

**Table 1:** Formulae for the rules of behavior of participants.

No.	Equation	Annotation
**Formula 1**	$△O=\frac{\sum_{A=1}^{N}O_{A2}-\sum_{A=1}^{N}O_{A1}}{N}$	△O represents the total amount of change in the opportunistic behavior tendency of all participants.
**Formula 2**	$P(A,B)=\frac{\frac{1}{D_{AB}}}{\sum_{B=1}^{N}{\frac{1}{D}}_{AB}}\;(A\neq B)$	D_AB_ refers to the closeness degree between two participants A and B connected by established trust relationship.
**Formula 3**	$△O(A,B)=K\frac{O_{B}}{D_{AB}}\;(A\neq B)$	O_B_ represents the immediate opportunistic behavior implementation tendency value of participant B.
**Formula 4**	$P_{link-other}=\frac{D_{AB}}{\sum_{B=1}^{N}D_{AB}}\;(A\neq B)$	Value range of Formula 3 is applicable.

According to social network theory, each participant can influence the implementation tendency of opportunistic behaviours on other participants who have established trust relationships. In fact, there is a probability (P(A,B)) that the two participants who have established trust relationships will influence each other, thus changing their opportunistic behaviour implementation tendency values. D_AB_ represents the closeness of the trust relationship between A and B, which is measured by the distance difference between the two coordinates in the simulation program [30]. This process is shown in Formula 2 of Table 1.

The opportunistic behavior implementation tendency of each participant will change with the progress of the project, so the participants in the trust networks will not only consider their own subjective will when making individual behavior decisions, but also comprehensively examine the networks in which they are embedded [31]. This concept of embeddedness can explain the influence of external environment on the behavior of the participant. In the trust networks, the participant B with low implementation tendency of opportunistic behavior changes the behavior tendency to the other participant A by relying on trust relationship connection (△O(A,B)). Previous study have shown that trust relationship in engineering projects has a significant negative impact on opportunism, and the coefficient K represents the influence of participant B on the opportunistic behavior tendency of the other participant A (K = -0.295) [19]. This influence mechanism is triggered if and only if participant A has a higher tendency to implement opportunistic behavior than any neighbor B in the trust network. This process is shown in Formula 3 in Table 1.

As the project progresses, it is possible to establish a new relationship of trust between the various participants. Conversely, existing trust relationships may be damaged and broken. Then there is a probability that a new trust relationship will appear between two participants (P_link-other_) that are not connected by trust relationship in the network [32]. This process is shown in Formula 4 in Table 1. On the other hand, there is no accurate quantitative method to quantify the damage and fracture of trust relationship in the network structure between the participants involved in hydraulic infrastructure projects, so this factor is set as a probability threshold value of 5% in this study [33]. That is, any relationship connection in the trust networks has a 5% probability of breaking during each simulation run, and the relationship breaking stops when the number of trust relationship connections in the network reaches the maximum possible value. At this time, each participant in the trust networks is fully trusted and the relationship is stable.

In general, this study uses simulation technology to simulate the trust networks environment of the project participants, which can form a large number of different trust network structures in a short time. The △O calculation result in this process has various possibilities, that is, it indicates that the trust network may inhibit the opportunistic behavior, or it may have no inhibitory effect at all. If the value of △O is negative, it indicates that the participant’s behavior tendency has been inhibited. The absolute value of △O indicates the degree of inhibition. In this study, the absolute value of △O is included in the regression analysis, that is, to explore the mechanism of trust networks and the degree of inhibition of opportunistic behavior tendency.

### Data acquisition

The initialisation routine of this study includes the initial setting of the network structure form, the number of nodes, the initial value of opportunistic behaviour implementation tendency, and the node coordinate value. Without loss of generality, the initial value of the number of trust network nodes is calculated as 30, representing each participant in the hydraulic infrastructure project. A unique trust network structure is generated during each simulation model process (Setup), and then the simulation program runs several times. Each time, a set of data combinations of trust network density, centralisation, and the variation of opportunistic behaviour implementation tendency (△O) are output. After executing the simulation program several times, it can gradually enter the stable state of trust networks; that is, the number of new and broken trust connections in the network is relatively stable. This process simulates the actual hydraulic infrastructure period and project progress. The output data of the trust network structure simulation operation in the initial state, running state, and stable state are recorded successively.

After running the simulation program, we can get some specific index data under the trust network state, including the trust network and the center potential as well as the implementation tendency and inhibition degree of opportunistic behavior. Each time the program runs to a stable state, about 50–70 sets of data are obtained. Therefore, it was necessary to repeat the above procedure and collect the dynamic change data combination obtained in the process, and finally obtain 1596 valid data combinations. After integrating the simulation programs and data collection, a database containing the simulation output data of several trust network models can be constructed, and the analysis can be carried out from the statistical significance to observe the change in individual behaviour and the emergence of overall behaviour characteristics in the trust networks. The trust network simulation model constructed in this study is dynamic; that is, each simulation model establishes a unique trust network structure model, which effectively reflects the complexity and heterogeneity of hydraulic infrastructure projects.

## Analysis and results

**Fig 2:**
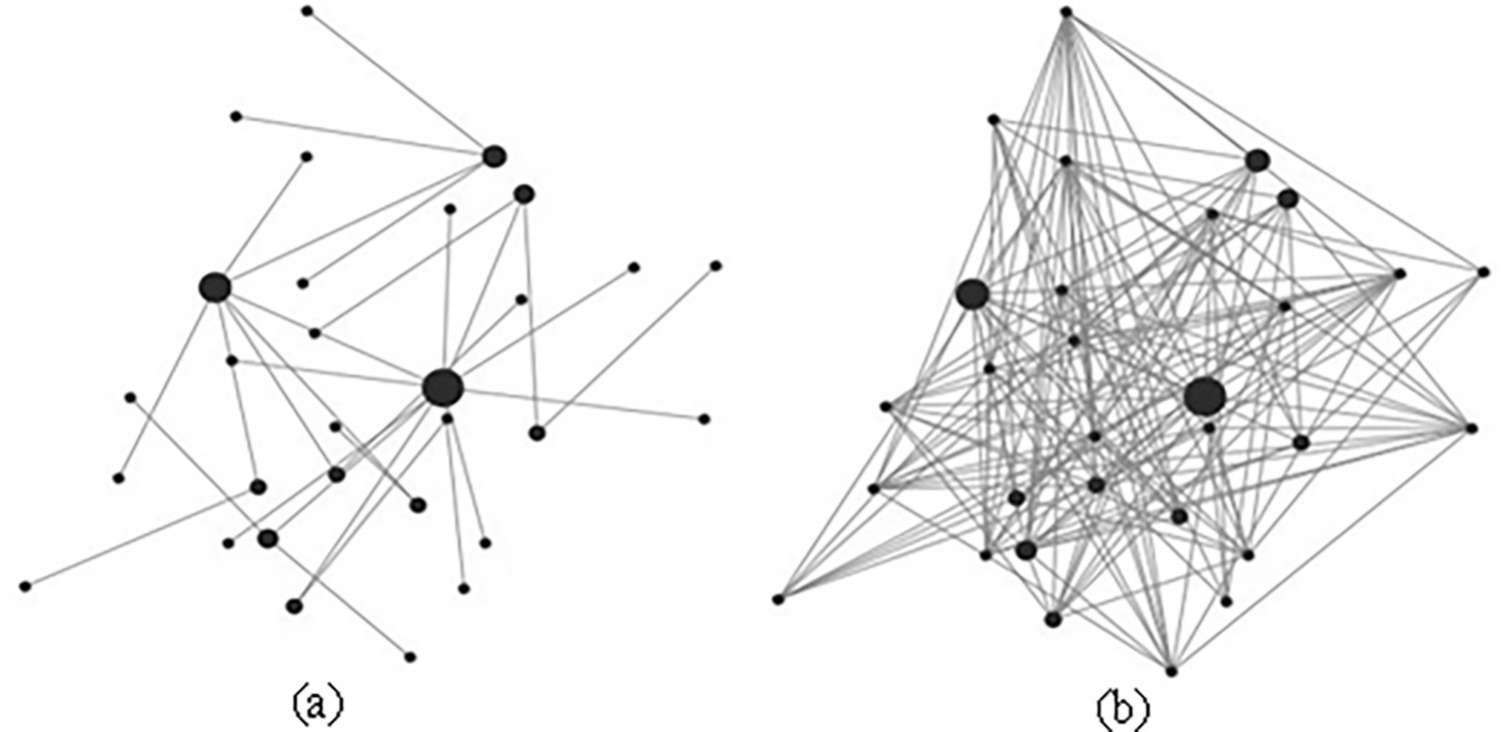
Changes of trust networks during simulation.

Program coding is carried out in NetLogo 6.2.0 platform, and the complete program code is detailed in the Appendix 1. On this basis, initialization routines are performed and trust relationship network is constructed, as shown in Fig 2A. Then through the continuous execution of the simulation program, the number of trust relationship connections in the network structure changes and eventually tends to be relatively stable, as shown in Fig 2B.

Each randomly generated trust network model can gradually enter a relatively stable state in the process of multiple iterations, but the number of iterations required is not the same. Then each randomly generated trust network structure can output different data scale. Therefore, this study selected the data of 50 trust network models that were randomly generated continuously to collect the data indicators before the model entered the stable state. The result is a database containing 1596 pieces of data.

The SPSS 24.0 platform is used for correlation analysis of these data, in order to test the correlation and linear relationship between the density, centralization and opportunistic behavior of the trust network formed by each participant in the project. The data obtained in this study show that △O are all negative values, which indicates that the opportunist behavior tendency is inhibited in the iterative process of the trust network. The degree of inhibition is expressed as the absolute value of △O. If the variation of opportunistic behavior tendency is large (△O), it indicates that the participator’s behavior in the trust networks is suppressed to a large extent. On the contrary, if the amount of behavior change is small, it indicates that the opportunistic behavior in the trust networks environment has not been inhibited.

### Density and opportunistic behaviour

**Fig 3:**
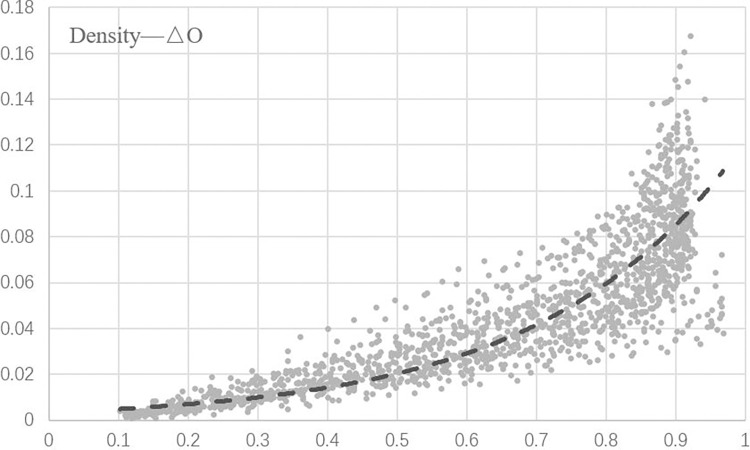
Scatter plot of density-△O output data.

Based on the collected data, we can tentatively judge that there is a correlation between the density of the trust networks and the change in the tendency of each participant to behave opportunistically. On this basis, scatter plots are drawn for the obtained data, as shown in Fig 3.

It can be judged that our data are linear, continuous and normally distributed, so we use Pearson correlation coefficient to test the correlation between variables, and the test results are shown in Tables 2–4.

**Table 2:** Designated items of self-service sampling (Density -△O).

Item	Result
Sampling method	Simple
Number of samples	2000
Confidence interval level	95.0%
Confidence interval type	Percentile

**Table 3:** Descriptive statistics of data (Density -△O).

Item		Result	Self-help sampling^a^		
			Deviation	95% Confidence interval	
				Lower limit	Upper limit
**Density**	Number of cases	1596	0	1596	1596
	Average	0.630	-0.000	0.618	0.642
	Standard deviation	0.246	-0.000	0.240	0.253
|**△O**|	Number of cases	1596	0	1596	1596
	Average	0.046	-0.000	0.045	0.048
	Standard deviation	0.030	-0.000	0.029	0.031

a. Self-sampling results are based on 2000 samples unless otherwise noted.

**Table 4:** Correlation analysis from parametric statistics (Density -△O).

Item				Density	△O
**Density**	Pearson correlation coefficient			1	0.885**
	Significance (two tailed)			/	0.000
	Number of cases			1596	1596
	Self-help sampling^b^	Deviation		0	0.000
		Standard deviation		0	0.006
		95% Confidence interval	Lower limit	1	0.873
			Upper limit	1	0.898
|**△O**|	Pearson correlation coefficient			0.885**	1
	Significance (two tailed)			0.000	/
	Number of cases			1596	1596
	Self-help sampling^b^	Deviation		0.000	0
		Standard deviation		0.006	0
		95% Confidence interval	Lower limit	0.873	1
			Upper limit	0.898	1

**. Indicates a significant correlation at the level of 0.01 (two-tailed).

b. Self-service sampling are based on 2000 samples unless otherwise stated.

Based on the data analysis results (Tables 2–4), it can be inferred that there is a positive correlation between trust networks density and the tendency to implement opportunistic behavior. The Pearson test yielded a correlation coefficient of 0.885, indicating a significant relationship. These findings suggest that an increase in trust networks density effectively restrains opportunistic behavior.

### Centralization and opportunistic behaviour

**Fig 4:**
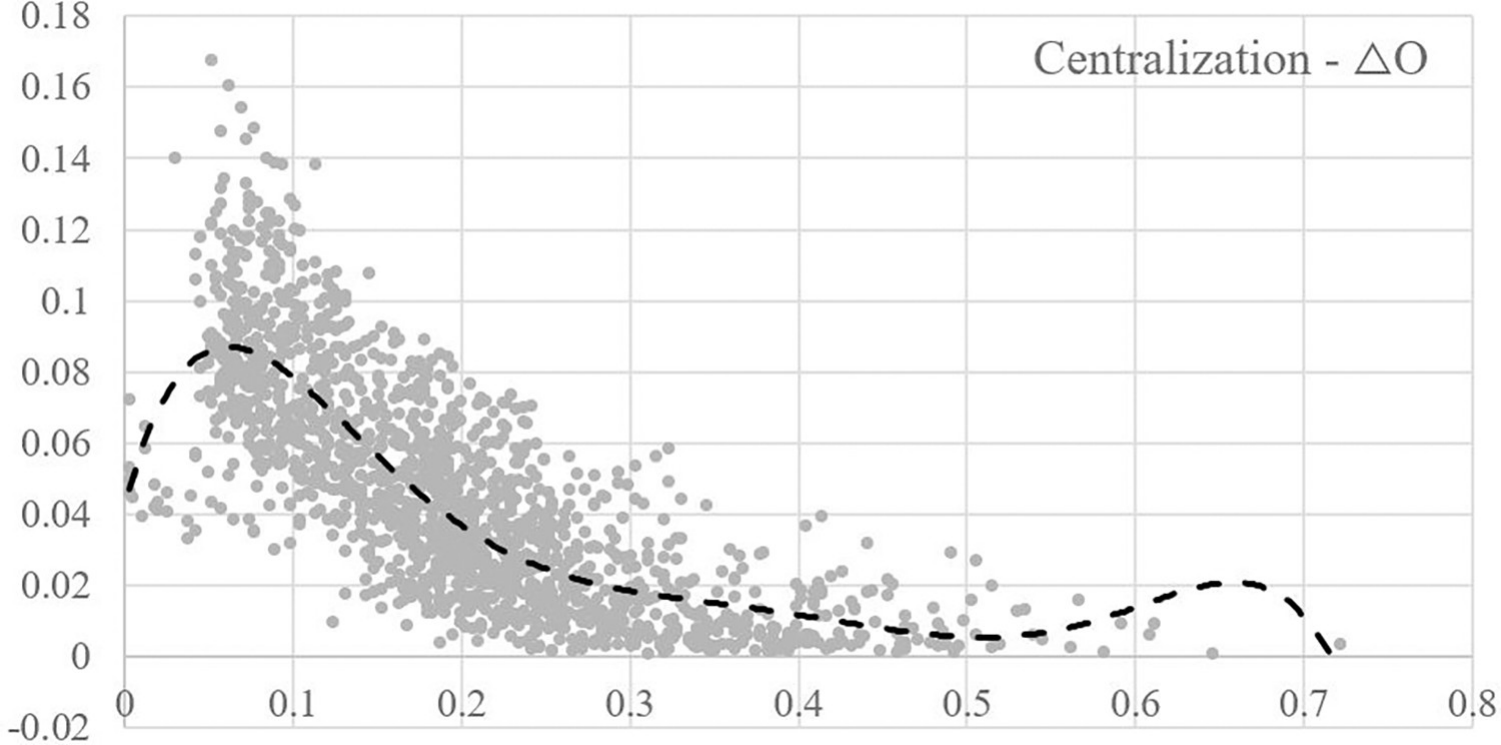
Scatter plot of centralization -△O output data.

The output data of modeling and simulation research cannot directly reflect the correlation between the centralization of trust networks and the variation of opportunistic behavior implementation tendency. Therefore, 1596 pieces of data in the database were first used to draw a scatter plot, to preliminarily fit the trend line between the two variables and intuitively reflect the correlation trend, as shown in Fig 4.

According to the correlation trend between the two variables shown in Fig 4, the correlation between the centralization of trust networks and the change in the tendency value of opportunistic behavior implementation is not clear, which requires further analysis and judgment. Therefore, 1596 data were analyzed using SPSS 24.0 platform. The specific data analysis process aligns with Research Methodology section of this study. Based on the data analysis results, it can be inferred that the degree of centralization in the trust networks is inversely correlated with changes in opportunist behavior implementation tendency. Specifically, the Pearson correlation coefficient obtained is -0.778, indicating a significant correlation at the 0.01 level. This suggests that as the degree of centralization in the trust networks increases, there is a decrease in opportunist behavior implementation tendency.

### Linear regression analysis

The variation of the implementation tendency of opportunistic behavior of each participant in the project is the dependent variable (Y:△O), and the independent variable is the density (X1) and centralization (X2) of the trust networks. On this basis, SPSS 24.0 software was used to conduct multiple linear regression analysis, and relevant data such as regression coefficients were obtained as shown in Table 5.

**Table 5:** Regression coefficientsa.

Model		Unstandardized Coefficients		Standardized Coefficients	T	Sig.	Collinearity Statistics
		B	Standard Error	Beta			VIF
**1**	**(Constant)**	-0.008	0.003		-3.005	0.003	
	**Density**	0.098	0.003	0.788	37.026	0.000	3.404
	**Centralization**	-0.034	0.006	-0.116	-5.435	0.000	3.404

R^2^: 0.788, Adjusted R^2^: 0.788, F = 2958.793 P = 0.000***.

a. Dependent variable: |△O|

b. Predictor variable: (Constant), Density, Centralization

***. Indicates a significant correlation at the level of 1%.

The multiple regression linear analysis model can be obtained from the above table: Y = 0.098X1–0.034X2–0.008. The density of trust networks of each construction participants is directly proportional to the change of the implementation tendency of opportunistic behaviors, that is, it can promote each construction participants to restrain its implementation tendency of opportunistic behaviors. However, the centralization of the trust networks of each construction participants is negatively correlated with the change of the implementation tendency of opportunistic behaviors, that is, it is difficult to promote each construction participants to restrain its implementation tendency of opportunistic behaviors. Based on the above, multiple tests were carried out to confirm the goodness of fit (Gof) of the regression equation, the significance of the regression equation and parameters, and the multicollinearity between variables, etc.

In fact, the Gof of the regression equation can be seen from the data analysis results in Table 5, that is, the degree of linear correlation between independent variables and dependent variables is determined. Specifically, R^2^ and adjusted R^2^ are 0.788, indicating a good degree of fitting, and it can be considered that explanatory variables have a good explanatory ability to the explained variables. On this basis, T-test can be performed for each parameter. It can be seen from Table 5 that the T values of the three regression coefficients are -3.005, 37.026 and -5.435 respectively. Therefore, it can be considered that each regression coefficient of the model obtained in this study has good significance. At the same time, it can be judged from the tolerance and VIF (variance inflation factor) data indexes in Table 5 that there is no collinearity problem among variables in this study.

In conclusion, the 1596 sets of data obtained through modeling and simulation analysis in this study can effectively build the regression model between the density of trust networks, the centralization and the variation of opportunistic behavior of each participant in the project. Among them, the density of trust networks of each participant is directly proportional to the change of implementing tendency of opportunistic behavior, while the centralization of trust networks of each participant is negatively related to the change of implementing tendency of opportunistic behavior.

## Discussion and implications

### Influence mechanism discussion

Trust networks formed by each participant in the hydraulic infrastructure project are in the process of dynamic evolution. Simulation modelling and data analysis can verify the influence of this network structure on opportunistic behaviour. A total of 1596 sets of data were collected, and each set of data has a unique variation of opportunistic behaviour (△O), which represents the degree of inhibition of opportunistic behaviour; the greater the value of the variation, the greater the degree of inhibition of behaviour. Simultaneously, the density and centralisation indexes corresponding to behavioural changes are output for each group of data. The results of the data analysis show that with an increase in trust network density, the implementation tendency of each participant’s opportunistic behaviour presents a significant downward trend. This shows that when the trust network density is large, the opportunistic behaviour of each participant in the hydraulic infrastructure project is restrained. On the contrary, with the increase in centralisation, the decrease in the implementation tendency of the opportunistic behaviour of each participant decreases. This indicates that when the centralisation of trust networks is high, the opportunistic behaviours of participants cannot be effectively suppressed.

Considering the influence mechanism revealed in this study from a deeper dimension, it can echo the classic argument put forward by economic sociologists. Economic activities and participants’ behaviors are influenced by the social environment in which they live. This study found that with the increase of trust networks density of participants, the implementation tendency of opportunistic behaviors decreased. In other words, it can be considered that high intensity trust networks density inhibits opportunistic behaviors. In this process, the trust network is a kind of social atmosphere, and the high intensity density index reflects the high degree of mutual trust among the participants. It should be noted that an increase in the density of the trust network does not necessarily lead to an increase or decrease in the centralisation indicator. The two indicators are independent of each other. On this premise, the centralisation of trust network reflects the concentration degree of a trust relationship. Therefore, a higher centralisation indicator presents a state in which there are both generally trusted and untrusted participant. The social phenomenon reflected by this state is the imbalance of the atmosphere of trust relationship, which may induce the untrusted to carry out opportunistic behavior. In view of the above, it is necessary to set up an institutional arrangement in the engineering project to promote the improvement of the density of the trust network and restrain the rise of the centralisation of the trust network by opening up communication channels and setting up information sharing platforms.

### Research results application

Previous study on management and sociology often involves the comprehensive application of a variety of research methods, among which the traditional methods are empirical or case analyses based on cross-sectional data [34]. However, these traditional methods cannot properly simulate the dynamic evolution of social network structures. To solve this problem, computer-aided simulation and modelling have become a better choice [35]. This study used the NetLogo platform to construct a trust network structure suitable for each participant in the hydraulic infrastructure project, and its influence on the implementation tendency of each participant’s opportunistic behaviour was analysed. Based on the complete code recorded in Appendix 1 of this study, the influence of trust network size, relationship strength, and other variables on the behaviour of the participant can be separately analysed, which is one of the academic innovations of this study.

Furthermore, hydraulic infrastructure projects present a network structure with the owner and construction contractor as the dual core, and the scale-free network model can better imitate this structure form. However, robustness and vulnerability are important characteristics of scale-free networks; that is, the existence of power-law distribution characteristics can promote the enhancement of centralisation. In fact, owners and construction contractors are in the structural holes of the networks and have a high centrality, so their opportunistic behaviours are more likely to overturn the trust atmosphere of the entire network organisation. Thus, the trust relationship between the construction participants is damaged on a large scale, that is, the density of the trust network is greatly reduced.

Combined with the data analysis results of this study, management inspiration and ideas can be provided from the perspective of resource and information integration; that is, integrated delivery mode (IPD) or whole-process engineering consulting mode can be used in engineering projects, that is, to break down the traditional dual-core network structure. Based on this, a geological survey, engineering design, engineering supervision, cost management, material and equipment procurement, and other businesses are integrated, and a centralised office system is implemented. In this process, the participants establish trust or consolidate the original relationship and reduce the edge nodes of the network structure that have fewer resources and information.

## Conclusions

Previous study on trust relationships and the impact on opportunistic behaviours tends to start from the binary perspective of project owners and contractors. However, this viewpoint is problematised with the concept of network-level trust. To solve this problem, this study reveals the relationship between trust networks and opportunistic behaviour among all participants in hydraulic infrastructure projects and fills in the research gaps.

The theoretical significance of this study is reflected in this, that is, it reveals the mechanism of the dynamic evolution of the participant trust network on the suppression of opportunistic behavior. The revelation of this mechanism can help researchers to clarify the extent to which the dynamic trust network influences the opportunistic behavior of each participant. In terms of practical significance, the conclusions of this study can help the project owners to formulate targeted project management strategies, and restrain the rapid growth of the centralisation while enhancing the density of trust networks. Specifically, based on the agent-based simulation and modelling method, a trust network simulation model suitable for hydraulic infrastructure projects was constructed, and a dynamic evolution analysis was implemented on this basis. The analysis results of this study can provide insight into the efficient cooperation and effective management of all participants in hydraulic infrastructure projects.

Based on the above, we provide a new theoretical exploration path for future researchers. The traditional research on project management only seems to focus on the binary relationship between the employer and the contractor, and carry out a lot of research on the project contract signed by the two before. However, due to the lack of effective management means or system design to regulate each participant of the project, opportunistic behavior spreads and accumulates among non-major contractors, which seriously affects the project performance. It is expected that based on the conclusion of this study, more management methods or regulations for project participants will emerge in the future, so as to manage engineering projects more comprehensively and realize the value of projects.

## Supporting information

S1 File Simulation modeling code. (PDF)

S2 File Experimental data recording. (PDF)
