# Supplementary material for: Agent-based simulation of trust networks and opportunistic behaviours of hydraulic infrastructure project participants
Source: PLoS One. 2025 Jan 6;20(1):e0316992. doi: 10.1371/journal.pone.0316992 (PMC11702997; doi:10.1371/journal.pone.0316992)
Supplement: S1 File — (PDF) [file pone.0316992.s001.pdf]

Appendix 1. Simulation modeling code

```
turtles-own
[
  last-o-behavior
 o-behavior
]
to setup
  clear-all
  setup-turtles
  setup-turtle-value
  reset-ticks
end

to go
  update-turtles-value
  setup-link-other
  setup-dislink-other
  tick
end

to setup-link-other
  ask turtles
  [       let sum-distance 0
    ask other turtles with [not link-neighbor? myself]
    [
     set sum-distance sum-distance +   compute-distance self myself
    ]
   ask other turtles with [not link-neighbor? myself]
    [
     if random-float 1 <(   compute-distance self myself / sum-distance)
      [
       create-link-with myself
      ]
    ]
```

```
  ]
end

to setup-dislink-other
ask links
  [
        if random-float 1 < dislink-other-probability and Density < 1
        [
          die
      ]
  ]
end

to-report Centralization
  let N count turtles
  report (max [count link-neighbors] of turtles * N    - sum [count link-neighbors] of turtles ) / (N
* N - 3 * N + 2)
end

to-report Density
  let N count turtles
  report count links * 2 /    (count turtles * count turtles - 1)
end

to update-turtles-value
  ask turtles
      [
      let sum-change-o-behavior 0
        let A-o-behavior o-behavior
        let sum-distance 0
        ask link-neighbors
        [
          set sum-distance sum-distance + 1 / compute-distance self myself
        ]
```

```
      ask link-neighbors
      [
        let probability    1 / compute-distance self myself    / sum-distance
        ; show probability
       if    o-behavior < A-o-behavior and    random-float 1 < probability
        [
         let Dab    compute-distance self myself
         set sum-change-o-behavior sum-change-o-behavior + k *    (o-behavior / Dab )
        ]
      ]
  ;    set last-o-behavior o-behavior
    set o-behavior o-behavior + sum-change-o-behavior
     ; show sum-change-o-behavior
  ]
end

to setup-turtle-value
  ask turtles [
  set    o-behavior precision (random-float 1) 10
  set    last-o-behavior o-behavior
  ]
end

to setup-turtles
  set-default-shape turtles "circle"
  ;; create two turtles (nodes) and space them out
  create-turtles 2 [
    set color yellow
    fd 5
  ]
  ask turtle 0 [ create-link-with turtle 1 ]    ;; create a link between them

  repeat ( num-nodes - 2 )
  [
```

```
   let partner one-of [ both-ends ] of one-of links
  create-turtles 1 [
    set color yellow
    move-to partner
    fd 2
    create-link-with partner
  ]
  layout
  ]
end

to-report compute-distance [turtle1 turtle2]
  let xcor1 [xcor] of turtle1
  let xcor2 [xcor] of turtle2
  let ycor1 [ycor] of turtle1
  let ycor2 [ycor] of turtle2
  report sqrt ((xcor1 - xcor2) ^ 2 + (ycor1 - ycor2)^ 2      )
end

to layout
  ask turtles [
  set size sqrt count my-links ]
  layout-spring turtles links 2 20 5
  ask turtles [
    facexy 0 0
    fd (distancexy 0 0) / 100
  ]
end
```
