# Supplementary material for: Agent-based simulation of trust networks and opportunistic behaviours of hydraulic infrastructure project participants
Source: PLoS One. 2025 Jan 6;20(1):e0316992. doi: 10.1371/journal.pone.0316992 (PMC11702997; doi:10.1371/journal.pone.0316992)
Supplement: S2 File — (PDF) [file pone.0316992.s002.pdf]

Appendix 2.The original records of 1596 experimental data in the study

Serial number	Density	Centralization	△O
1	0.142	0.286	-0.005
2	0.2	0.259	-0.008
3	0.245	0.246	-0.013
4	0.325	0.268	-0.016
5	0.38	0.244	-0.018
6	0.458	0.232	-0.023
7	0.512	0.246	-0.028
8	0.598	0.224	-0.03
9	0.663	0.227	-0.032
10	0.765	0.224	-0.034
11	0.816	0.167	-0.037
12	0.874	0.103	-0.039
13	0.921	0.052	-0.043
14	0.952	0.017	-0.048
15	0.14	0.362	-0.002
16	0.194	0.34	-0.006
17	0.234	0.369	-0.009
18	0.311	0.357	-0.012
19	0.387	0.31	-0.016
20	0.438	0.291	-0.019
21	0.498	0.261	-0.021
22	0.565	0.298	-0.025
23	0.636	0.256	-0.027
24	0.721	0.2	-0.031
25	0.788	0.163	-0.034
26	0.839	0.143	-0.034
27	0.901	0.074	-0.039
28	0.945	0.025	-0.041
29	0.966	0.002	-0.046
30	0.12	0.347	-0.002
31	0.174	0.325	-0.005
32	0.249	0.352	-0.008
33	0.331	0.446	-0.01
34	0.407	0.399	-0.014
35	0.463	0.374	-0.018
36	0.516	0.315	-0.024
37	0.594	0.377	-0.029
38	0.672	0.328	-0.033
39	0.734	0.259	-0.036
40	0.783	0.204	-0.041
41	0.843	0.138	-0.047
42	0.894	0.081	-0.052
43	0.957	0.012	-0.059
44	0.122	0.382	-0.004
45	0.194	0.377	-0.006
46	0.26	0.34	-0.007
47	0.318	0.276	-0.009
48	0.389	0.234	-0.011
49	0.465	0.187	-0.016
50	0.527	0.303	-0.019
51	0.598	0.261	-0.022
52	0.65	0.241	-0.024
53	0.701	0.185	-0.026
54	0.77	0.145	-0.028
55	0.83	0.153	-0.033
56	0.899	0.076	-0.035
57	0.95	0.02	-0.043
58	0.966	0.002	-0.053
59	0.109	0.397	-0.003
60	0.178	0.394	-0.007
61	0.222	0.345	-0.01
62	0.26	0.229	-0.011
63	0.305	0.18	-0.012
64	0.334	0.259	-0.015
65	0.372	0.18	-0.017
66	0.396	0.153	-0.019
67	0.44	0.177	-0.02
68	0.474	0.214	-0.024
69	0.529	0.19	-0.027
70	0.529	0.227	-0.031
71	0.563	0.19	-0.033
72	0.59	0.197	-0.036
73	0.614	0.207	-0.038
74	0.681	0.207	-0.042
75	0.694	0.229	-0.046
76	0.725	0.195	-0.05
77	0.716	0.241	-0.052
78	0.75	0.204	-0.056
79	0.768	0.222	-0.06
80	0.781	0.207	-0.062
81	0.794	0.155	-0.064
82	0.814	0.17	-0.067
83	0.812	0.172	-0.068
84	0.814	0.17	-0.074
85	0.832	0.15	-0.078
86	0.863	0.116	-0.083
87	0.113	0.318	-0.005
88	0.149	0.352	-0.007
89	0.189	0.345	-0.009
90	0.24	0.251	-0.012
91	0.291	0.195	-0.016
92	0.325	0.268	-0.019
93	0.367	0.222	-0.022
94	0.414	0.207	-0.026
95	0.443	0.175	-0.028
96	0.498	0.261	-0.034
97	0.536	0.182	-0.037
98	0.583	0.204	-0.04
99	0.625	0.232	-0.045
100	0.663	0.227	-0.046
101	0.687	0.2	-0.051
102	0.71	0.212	-0.054
103	0.741	0.214	-0.06
104	0.763	0.153	-0.065
105	0.805	0.18	-0.068
106	0.796	0.153	-0.07
107	0.814	0.17	-0.075
108	0.828	0.118	-0.077
109	0.832	0.15	-0.079
110	0.841	0.14	-0.083
111	0.868	0.111	-0.086
112	0.877	0.101	-0.09
113	0.879	0.062	-0.092
114	0.897	0.079	-0.098
115	0.129	0.411	-0.004
116	0.189	0.345	-0.007
117	0.231	0.261	-0.013
118	0.296	0.3	-0.018
119	0.354	0.273	-0.021
120	0.407	0.214	-0.027
121	0.436	0.219	-0.032
122	0.472	0.254	-0.037
123	0.514	0.244	-0.041
124	0.536	0.33	-0.044
125	0.576	0.323	-0.049
126	0.616	0.315	-0.056
127	0.643	0.323	-0.059
128	0.652	0.239	-0.066
129	0.716	0.241	-0.071
130	0.754	0.163	-0.075
131	0.765	0.187	-0.079
132	0.776	0.175	-0.081
133	0.803	0.182	-0.083
134	0.808	0.177	-0.089
135	0.823	0.16	-0.091
136	0.85	0.131	-0.1
137	0.854	0.126	-0.102
138	0.879	0.099	-0.115
139	0.89	0.086	-0.122
140	0.919	0.054	-0.125
141	0.914	0.059	-0.134
142	0.883	0.094	-0.138
143	0.118	0.35	-0.003
144	0.194	0.377	-0.007
145	0.24	0.251	-0.008
146	0.291	0.232	-0.012
147	0.334	0.148	-0.018
148	0.383	0.167	-0.02
149	0.409	0.249	-0.028
150	0.469	0.182	-0.034
151	0.516	0.241	-0.039
152	0.57	0.219	-0.045
153	0.605	0.18	-0.053
154	0.659	0.195	-0.059
155	0.699	0.224	-0.062
156	0.73	0.227	-0.071
157	0.761	0.192	-0.076

158	0.776	0.175	-0.083
159	0.794	0.192	-0.084
160	0.801	0.148	-0.09
161	0.816	0.131	-0.093
162	0.843	0.101	-0.103
163	0.836	0.145	-0.108
164	0.874	0.103	-0.12
165	0.879	0.099	-0.129
166	0.888	0.089	-0.139
167	0.903	0.071	-0.145
168	0.899	0.076	-0.149
169	0.912	0.062	-0.161
170	0.921	0.052	-0.168
171	0.111	0.32	-0.002
172	0.156	0.345	-0.003
173	0.211	0.32	-0.005
174	0.278	0.283	-0.007
175	0.334	0.296	-0.01
176	0.392	0.268	-0.012
177	0.438	0.254	-0.014
178	0.474	0.251	-0.015
179	0.525	0.195	-0.017
180	0.547	0.17	-0.021
181	0.601	0.185	-0.022
182	0.632	0.15	-0.027
183	0.667	0.185	-0.03
184	0.727	0.192	-0.034
185	0.752	0.128	-0.038
186	0.812	0.172	-0.041
187	0.816	0.131	-0.043
188	0.85	0.094	-0.047
189	0.865	0.113	-0.052
190	0.885	0.091	-0.054
191	0.883	0.094	-0.057
192	0.899	0.076	-0.059
193	0.908	0.067	-0.066
194	0.903	0.071	-0.07
195	0.894	0.081	-0.075
196	0.899	0.076	-0.076
197	0.899	0.076	-0.08
198	0.899	0.076	-0.085
199	0.109	0.544	-0.005
200	0.185	0.498	-0.01
201	0.218	0.424	-0.013
202	0.278	0.431	-0.016
203	0.303	0.441	-0.018
204	0.34	0.399	-0.021
205	0.407	0.362	-0.022
206	0.472	0.365	-0.028
207	0.503	0.33	-0.033
208	0.543	0.249	-0.037
209	0.561	0.229	-0.044
210	0.63	0.264	-0.047
211	0.67	0.293	-0.049
212	0.701	0.222	-0.052
213	0.743	0.212	-0.056
214	0.77	0.145	-0.061
215	0.792	0.195	-0.065
216	0.828	0.155	-0.068
217	0.861	0.118	-0.073
218	0.89	0.086	-0.079
219	0.917	0.057	-0.087
220	0.897	0.079	-0.09
221	0.89	0.086	-0.093
222	0.881	0.096	-0.101
223	0.892	0.084	-0.108
224	0.89	0.086	-0.11
225	0.903	0.071	-0.117
226	0.91	0.064	-0.12
227	0.125	0.342	-0.004
228	0.174	0.362	-0.007
229	0.234	0.369	-0.012
230	0.285	0.276	-0.013
231	0.309	0.212	-0.017
232	0.354	0.163	-0.021
233	0.392	0.158	-0.025
234	0.449	0.204	-0.026
235	0.496	0.19	-0.029
236	0.545	0.172	-0.032
237	0.565	0.224	-0.035
238	0.583	0.241	-0.038
239	0.621	0.2	-0.04
240	0.659	0.195	-0.048
241	0.727	0.229	-0.05
242	0.736	0.182	-0.053
243	0.774	0.14	-0.055
244	0.796	0.19	-0.059
245	0.796	0.19	-0.067
246	0.812	0.172	-0.068
247	0.823	0.16	-0.073
248	0.83	0.153	-0.077
249	0.83	0.116	-0.081
250	0.845	0.135	-0.087
251	0.859	0.121	-0.093
252	0.872	0.106	-0.095
253	0.872	0.106	-0.098
254	0.885	0.091	-0.102
255	0.154	0.31	-0.001
256	0.196	0.3	-0.003
257	0.247	0.244	-0.007
258	0.314	0.244	-0.01
259	0.394	0.303	-0.012
260	0.434	0.296	-0.013
261	0.483	0.241	-0.015
262	0.534	0.222	-0.02
263	0.59	0.197	-0.024
264	0.596	0.19	-0.026
265	0.627	0.229	-0.03
266	0.674	0.214	-0.032
267	0.699	0.224	-0.035
268	0.725	0.195	-0.038
269	0.763	0.153	-0.04
270	0.779	0.135	-0.045
271	0.816	0.131	-0.049
272	0.834	0.148	-0.051
273	0.839	0.106	-0.052
274	0.87	0.108	-0.056
275	0.89	0.086	-0.06
276	0.874	0.103	-0.065
277	0.881	0.096	-0.067
278	0.894	0.081	-0.069
279	0.905	0.069	-0.072
280	0.91	0.064	-0.077
281	0.91	0.064	-0.08
282	0.899	0.076	-0.084
283	0.131	0.187	-0.004
284	0.189	0.234	-0.009
285	0.238	0.254	-0.013
286	0.305	0.217	-0.017
287	0.369	0.219	-0.02
288	0.436	0.219	-0.023
289	0.467	0.185	-0.025
290	0.503	0.145	-0.029
291	0.547	0.17	-0.034
292	0.581	0.17	-0.038
293	0.612	0.209	-0.043
294	0.647	0.17	-0.048
295	0.69	0.16	-0.051
296	0.734	0.185	-0.054
297	0.756	0.16	-0.061
298	0.792	0.158	-0.064
299	0.792	0.195	-0.07
300	0.814	0.17	-0.08
301	0.825	0.121	-0.083
302	0.83	0.153	-0.093
303	0.85	0.131	-0.102
304	0.857	0.123	-0.104
305	0.888	0.089	-0.107
306	0.885	0.091	-0.11
307	0.892	0.084	-0.118
308	0.89	0.086	-0.125
309	0.877	0.101	-0.127
310	0.865	0.113	-0.138
311	0.122	0.493	-0.002
312	0.16	0.488	-0.004
313	0.247	0.539	-0.006
314	0.3	0.591	-0.009
315	0.356	0.53	-0.013
316	0.414	0.502	-0.016
317	0.432	0.409	-0.018
318	0.478	0.32	-0.021
319	0.514	0.355	-0.024

320	0.554	0.31	-0.029
321	0.598	0.261	-0.037
322	0.645	0.209	-0.041
323	0.674	0.251	-0.046
324	0.701	0.185	-0.054
325	0.725	0.232	-0.058
326	0.765	0.224	-0.065
327	0.803	0.182	-0.072
328	0.81	0.175	-0.077
329	0.834	0.148	-0.085
330	0.839	0.143	-0.089
331	0.857	0.123	-0.093
332	0.861	0.118	-0.101
333	0.859	0.121	-0.107
334	0.888	0.089	-0.113
335	0.879	0.062	-0.116
336	0.885	0.091	-0.122
337	0.901	0.074	-0.128
338	0.901	0.074	-0.13
339	0.125	0.416	-0.002
340	0.185	0.424	-0.004
341	0.216	0.426	-0.005
342	0.242	0.397	-0.007
343	0.283	0.352	-0.009
344	0.32	0.236	-0.011
345	0.354	0.236	-0.013
346	0.38	0.207	-0.015
347	0.44	0.177	-0.016
348	0.476	0.323	-0.019
349	0.529	0.337	-0.022
350	0.57	0.219	-0.026
351	0.583	0.241	-0.028
352	0.623	0.234	-0.032
353	0.647	0.244	-0.035
354	0.67	0.219	-0.039
355	0.67	0.182	-0.04
356	0.703	0.219	-0.043
357	0.745	0.172	-0.047
358	0.768	0.185	-0.05
359	0.783	0.167	-0.052
360	0.801	0.148	-0.054
361	0.81	0.175	-0.06
362	0.843	0.138	-0.061
363	0.868	0.111	-0.064
364	0.861	0.081	-0.07
365	0.87	0.108	-0.073
366	0.883	0.094	-0.076
367	0.149	0.389	-0.002
368	0.202	0.367	-0.004
369	0.254	0.384	-0.006
370	0.309	0.36	-0.01
371	0.36	0.34	-0.012
372	0.418	0.313	-0.016
373	0.474	0.325	-0.018
374	0.523	0.308	-0.019
375	0.552	0.276	-0.025
376	0.57	0.256	-0.027
377	0.612	0.246	-0.032
378	0.654	0.236	-0.036
379	0.694	0.155	-0.039
380	0.732	0.15	-0.041
381	0.745	0.172	-0.043
382	0.763	0.19	-0.046
383	0.808	0.14	-0.047
384	0.825	0.158	-0.051
385	0.85	0.131	-0.056
386	0.861	0.081	-0.059
387	0.879	0.099	-0.062
388	0.899	0.076	-0.067
389	0.908	0.067	-0.071
390	0.912	0.062	-0.077
391	0.912	0.062	-0.081
392	0.908	0.067	-0.084
393	0.883	0.094	-0.086
394	0.877	0.064	-0.091
395	0.122	0.271	-0.002
396	0.182	0.278	-0.005
397	0.247	0.207	-0.008
398	0.307	0.288	-0.01
399	0.349	0.315	-0.012
400	0.392	0.305	-0.015
401	0.465	0.224	-0.018
402	0.516	0.241	-0.021
403	0.532	0.261	-0.025
404	0.59	0.234	-0.027
405	0.618	0.202	-0.029
406	0.625	0.195	-0.033
407	0.674	0.177	-0.036
408	0.723	0.16	-0.039
409	0.765	0.187	-0.042
410	0.799	0.15	-0.046
411	0.836	0.145	-0.05
412	0.85	0.131	-0.053
413	0.865	0.113	-0.058
414	0.859	0.121	-0.061
415	0.854	0.126	-0.064
416	0.861	0.118	-0.067
417	0.868	0.111	-0.07
418	0.897	0.079	-0.072
419	0.903	0.071	-0.076
420	0.917	0.057	-0.079
421	0.901	0.074	-0.081
422	0.917	0.057	-0.088
423	0.145	0.246	-0.003
424	0.194	0.229	-0.006
425	0.214	0.244	-0.008
426	0.274	0.214	-0.013
427	0.298	0.187	-0.019
428	0.343	0.249	-0.026
429	0.414	0.207	-0.035
430	0.461	0.155	-0.039
431	0.483	0.204	-0.044
432	0.512	0.209	-0.049
433	0.538	0.217	-0.055
434	0.585	0.239	-0.059
435	0.632	0.187	-0.063
436	0.654	0.2	-0.066
437	0.703	0.219	-0.069
438	0.732	0.224	-0.071
439	0.754	0.163	-0.073
440	0.761	0.192	-0.076
441	0.774	0.177	-0.08
442	0.799	0.187	-0.085
443	0.823	0.16	-0.088
444	0.854	0.126	-0.092
445	0.868	0.111	-0.093
446	0.877	0.101	-0.096
447	0.863	0.116	-0.099
448	0.872	0.106	-0.105
449	0.865	0.113	-0.111
450	0.89	0.086	-0.114
451	0.129	0.448	-0.002
452	0.202	0.404	-0.006
453	0.242	0.36	-0.011
454	0.305	0.401	-0.016
455	0.358	0.305	-0.02
456	0.407	0.251	-0.026
457	0.429	0.264	-0.029
458	0.474	0.251	-0.035
459	0.521	0.2	-0.038
460	0.556	0.197	-0.044
461	0.603	0.182	-0.048
462	0.627	0.118	-0.053
463	0.656	0.197	-0.059
464	0.672	0.18	-0.068
465	0.721	0.2	-0.072
466	0.743	0.138	-0.078
467	0.759	0.195	-0.082
468	0.788	0.163	-0.089
469	0.819	0.128	-0.093
470	0.854	0.089	-0.097
471	0.859	0.121	-0.105
472	0.877	0.064	-0.111
473	0.885	0.091	-0.117
474	0.892	0.084	-0.125
475	0.917	0.057	-0.132
476	0.941	0.03	-0.14
477	0.917	0.057	-0.148
478	0.905	0.069	-0.155
479	0.142	0.397	-0.004
480	0.194	0.377	-0.007
481	0.231	0.409	-0.015

482	0.291	0.453	-0.017
483	0.323	0.456	-0.02
484	0.383	0.426	-0.024
485	0.445	0.505	-0.027
486	0.492	0.49	-0.029
487	0.503	0.441	-0.032
488	0.536	0.404	-0.037
489	0.561	0.414	-0.039
490	0.59	0.345	-0.042
491	0.641	0.251	-0.046
492	0.692	0.268	-0.051
493	0.696	0.264	-0.057
494	0.741	0.214	-0.06
495	0.779	0.209	-0.064
496	0.821	0.163	-0.068
497	0.839	0.143	-0.071
498	0.859	0.121	-0.074
499	0.872	0.069	-0.078
500	0.885	0.091	-0.084
501	0.894	0.081	-0.086
502	0.903	0.071	-0.092
503	0.908	0.067	-0.097
504	0.917	0.057	-0.08153
505	0.91	0.064	-0.07934
506	0.908	0.067	-0.08214
507	0.122	0.271	0.007399
508	0.2	0.259	-0.00092
509	0.236	0.219	-0.00595
510	0.287	0.2	-0.01427
511	0.329	0.264	-0.01573
512	0.387	0.163	-0.02406
513	0.412	0.209	-0.02691
514	0.465	0.224	-0.0277
515	0.494	0.229	-0.03152
516	0.512	0.209	-0.03473
517	0.561	0.192	-0.04172
518	0.594	0.192	-0.0433
519	0.625	0.195	-0.04561
520	0.659	0.195	-0.04707
521	0.694	0.192	-0.05114
522	0.732	0.187	-0.05774
523	0.763	0.227	-0.06052
524	0.781	0.207	-0.05932
525	0.81	0.138	-0.06789
526	0.839	0.143	-0.06795
527	0.85	0.131	-0.07215
528	0.879	0.099	-0.07438
529	0.905	0.069	-0.07779
530	0.91	0.064	-0.07934
531	0.919	0.054	-0.08071
532	0.912	0.062	-0.08339
533	0.91	0.064	-0.08298
534	0.921	0.052	-0.08494
535	0.127	0.562	0.016475
536	0.2	0.406	0.005446
537	0.287	0.31	-0.00797
538	0.334	0.369	-0.01111
539	0.369	0.293	-0.01519
540	0.416	0.167	-0.02626
541	0.465	0.224	-0.02863
542	0.498	0.187	-0.03594
543	0.543	0.212	-0.03556
544	0.596	0.3	-0.039
545	0.594	0.266	-0.03937
546	0.607	0.214	-0.0435
547	0.627	0.192	-0.0489
548	0.654	0.236	-0.04987
549	0.696	0.19	-0.05666
550	0.734	0.185	-0.06113
551	0.77	0.182	-0.06263
552	0.808	0.14	-0.0684
553	0.823	0.16	-0.06971
554	0.854	0.126	-0.0698
555	0.85	0.131	-0.0696
556	0.861	0.081	-0.07863
557	0.859	0.121	-0.076
558	0.919	0.054	-0.08001
559	0.908	0.067	-0.08362
560	0.912	0.062	-0.08169
561	0.928	0.044	-0.08675
562	0.917	0.057	-0.07959

563	0.102	0.478	0.016716
564	0.149	0.463	0.009784
565	0.209	0.433	0.005719
566	0.26	0.414	-0.00035
567	0.329	0.411	-0.00591
568	0.369	0.367	-0.01275
569	0.42	0.31	-0.027
570	0.463	0.227	-0.033
571	0.512	0.32	-0.039
572	0.549	0.278	-0.042
573	0.578	0.283	-0.045
574	0.603	0.293	-0.052
575	0.625	0.232	-0.055
576	0.679	0.172	-0.058
577	0.687	0.163	-0.063
578	0.723	0.197	-0.066
579	0.741	0.214	-0.072
580	0.765	0.15	-0.074
581	0.779	0.135	-0.078
582	0.796	0.153	-0.084
583	0.83	0.116	-0.088
584	0.854	0.126	-0.098
585	0.865	0.113	-0.106
586	0.883	0.094	-0.116
587	0.905	0.069	-0.118
588	0.901	0.074	-0.122
589	0.903	0.071	-0.133
590	0.892	0.084	-0.14
591	0.12	0.495	-0.003
592	0.178	0.505	-0.006
593	0.229	0.411	-0.012
594	0.294	0.414	-0.018
595	0.34	0.362	-0.022
596	0.396	0.3	-0.029
597	0.447	0.207	-0.033
598	0.498	0.187	-0.036
599	0.541	0.251	-0.04
600	0.561	0.303	-0.044
601	0.565	0.298	-0.048
602	0.616	0.278	-0.051
603	0.661	0.303	-0.054
604	0.663	0.19	-0.057
605	0.685	0.165	-0.059
606	0.694	0.192	-0.061
607	0.741	0.214	-0.066
608	0.776	0.175	-0.072
609	0.819	0.165	-0.078
610	0.825	0.158	-0.083
611	0.841	0.103	-0.087
612	0.87	0.108	-0.092
613	0.859	0.121	-0.096
614	0.881	0.096	-0.1
615	0.905	0.069	-0.104
616	0.919	0.054	-0.106
617	0.91	0.064	-0.109
618	0.905	0.069	-0.114
619	0.12	0.273	-0.004
620	0.171	0.328	-0.008
621	0.238	0.254	-0.013
622	0.271	0.254	-0.017
623	0.347	0.281	-0.022
624	0.427	0.303	-0.025
625	0.469	0.256	-0.028
626	0.516	0.278	-0.031
627	0.552	0.239	-0.035
628	0.601	0.296	-0.039
629	0.65	0.278	-0.043
630	0.663	0.227	-0.045
631	0.696	0.227	-0.047
632	0.761	0.155	-0.05
633	0.761	0.192	-0.055
634	0.814	0.17	-0.058
635	0.83	0.153	-0.061
636	0.854	0.126	-0.067
637	0.859	0.121	-0.071
638	0.894	0.081	-0.075
639	0.905	0.069	-0.079
640	0.91	0.064	-0.084
641	0.919	0.054	-0.089
642	0.899	0.076	-0.094
643	0.928	0.044	-0.1

644	0.93	0.042	-0.106
645	0.921	0.052	-0.11
646	0.908	0.067	-0.114
647	0.118	0.35	-0.002
648	0.182	0.278	-0.003
649	0.242	0.286	-0.007
650	0.285	0.202	-0.009
651	0.331	0.224	-0.01
652	0.358	0.268	-0.017
653	0.418	0.202	-0.023
654	0.476	0.249	-0.03
655	0.521	0.31	-0.032
656	0.554	0.273	-0.035
657	0.614	0.207	-0.039
658	0.627	0.229	-0.041
659	0.661	0.229	-0.042
660	0.692	0.195	-0.045
661	0.727	0.192	-0.048
662	0.741	0.177	-0.052
663	0.745	0.172	-0.054
664	0.77	0.182	-0.056
665	0.801	0.185	-0.058
666	0.819	0.128	-0.065
667	0.845	0.135	-0.066
668	0.868	0.111	-0.07
669	0.885	0.091	-0.074
670	0.901	0.074	-0.076
671	0.903	0.071	-0.078
672	0.908	0.067	-0.081
673	0.908	0.067	-0.086
674	0.923	0.049	-0.09
675	0.131	0.483	-0.003
676	0.174	0.436	-0.004
677	0.238	0.438	-0.006
678	0.294	0.34	-0.008
679	0.336	0.293	-0.01
680	0.392	0.305	-0.013
681	0.434	0.296	-0.016
682	0.478	0.246	-0.018
683	0.503	0.219	-0.021
684	0.547	0.207	-0.023
685	0.614	0.207	-0.026
686	0.625	0.232	-0.028
687	0.643	0.175	-0.031
688	0.663	0.153	-0.032
689	0.703	0.145	-0.034
690	0.741	0.103	-0.037
691	0.75	0.131	-0.039
692	0.808	0.14	-0.041
693	0.834	0.148	-0.043
694	0.841	0.14	-0.045
695	0.865	0.113	-0.049
696	0.877	0.101	-0.052
697	0.888	0.089	-0.056
698	0.877	0.101	-0.061
699	0.883	0.094	-0.064
700	0.89	0.086	-0.068
701	0.914	0.059	-0.07
702	0.91	0.064	-0.072
703	0.127	0.34	-0.003
704	0.191	0.342	-0.006
705	0.236	0.293	-0.008
706	0.271	0.291	-0.01
707	0.354	0.2	-0.013
708	0.385	0.276	-0.014
709	0.438	0.291	-0.016
710	0.494	0.303	-0.018
711	0.536	0.219	-0.02
712	0.567	0.222	-0.021
713	0.598	0.187	-0.025
714	0.63	0.227	-0.028
715	0.661	0.229	-0.03
716	0.692	0.268	-0.031
717	0.719	0.202	-0.034
718	0.75	0.241	-0.038
719	0.77	0.182	-0.042
720	0.792	0.158	-0.046
721	0.808	0.14	-0.051
722	0.823	0.123	-0.053
723	0.845	0.135	-0.055
724	0.85	0.131	-0.057
725	0.87	0.108	-0.059
726	0.874	0.103	-0.061
727	0.859	0.084	-0.063
728	0.865	0.113	-0.066
729	0.85	0.094	-0.069
730	0.859	0.121	-0.07
731	0.118	0.645	-0.001
732	0.176	0.581	-0.001
733	0.236	0.515	-0.003
734	0.285	0.461	-0.004
735	0.343	0.47	-0.005
736	0.374	0.436	-0.006
737	0.407	0.288	-0.009
738	0.456	0.234	-0.01
739	0.487	0.236	-0.011
740	0.525	0.232	-0.012
741	0.563	0.153	-0.014
742	0.618	0.165	-0.016
743	0.672	0.143	-0.017
744	0.692	0.158	-0.021
745	0.712	0.172	-0.024
746	0.723	0.197	-0.029
747	0.736	0.182	-0.032
748	0.745	0.209	-0.034
749	0.741	0.214	-0.036
750	0.763	0.19	-0.039
751	0.794	0.192	-0.04
752	0.781	0.17	-0.041
753	0.816	0.167	-0.044
754	0.808	0.103	-0.045
755	0.83	0.116	-0.05
756	0.843	0.101	-0.055
757	0.839	0.143	-0.058
758	0.861	0.118	-0.062
759	0.118	0.35	-0.002
760	0.169	0.404	-0.004
761	0.222	0.271	-0.006
762	0.271	0.291	-0.008
763	0.309	0.212	-0.009
764	0.363	0.337	-0.012
765	0.432	0.261	-0.013
766	0.476	0.212	-0.015
767	0.541	0.251	-0.016
768	0.574	0.251	-0.017
769	0.607	0.251	-0.02
770	0.652	0.276	-0.022
771	0.687	0.273	-0.024
772	0.719	0.239	-0.026
773	0.761	0.192	-0.029
774	0.792	0.195	-0.032
775	0.812	0.172	-0.035
776	0.825	0.158	-0.037
777	0.854	0.126	-0.039
778	0.854	0.126	-0.04
779	0.857	0.086	-0.043
780	0.868	0.111	-0.045
781	0.897	0.079	-0.048
782	0.912	0.062	-0.051
783	0.923	0.049	-0.052
784	0.91	0.064	-0.054
785	0.93	0.042	-0.056
786	0.897	0.079	-0.057
787	0.149	0.463	-0.004
788	0.211	0.468	-0.008
789	0.276	0.433	-0.013
790	0.367	0.333	-0.017
791	0.432	0.335	-0.021
792	0.487	0.273	-0.024
793	0.552	0.239	-0.029
794	0.614	0.17	-0.034
795	0.683	0.167	-0.039
796	0.741	0.14	-0.045
797	0.803	0.182	-0.048
798	0.874	0.103	-0.053
799	0.93	0.042	-0.057
800	0.957	0.012	-0.065
801	0.966	0.002	-0.072
802	0.14	0.399	-0.004
803	0.205	0.328	-0.007
804	0.276	0.36	-0.01
805	0.365	0.335	-0.012

806	0.438	0.254	-0.015
807	0.505	0.254	-0.018
808	0.57	0.219	-0.023
809	0.654	0.236	-0.024
810	0.725	0.232	-0.028
811	0.794	0.192	-0.031
812	0.857	0.123	-0.034
813	0.91	0.064	-0.039
814	0.952	0.017	-0.042
815	0.963	0.005	-0.045
816	0.129	0.374	-0.002
817	0.196	0.337	-0.004
818	0.254	0.31	-0.008
819	0.345	0.283	-0.013
820	0.407	0.214	-0.016
821	0.476	0.212	-0.021
822	0.565	0.187	-0.022
823	0.621	0.163	-0.023
824	0.676	0.212	-0.029
825	0.756	0.197	-0.034
826	0.814	0.17	-0.038
827	0.865	0.113	-0.041
828	0.917	0.057	-0.042
829	0.945	0.025	-0.046
830	0.966	0.002	-0.05
831	0.14	0.362	-0.003
832	0.209	0.397	-0.006
833	0.291	0.305	-0.009
834	0.347	0.281	-0.012
835	0.418	0.202	-0.016
836	0.487	0.2	-0.018
837	0.549	0.204	-0.021
838	0.63	0.227	-0.022
839	0.714	0.17	-0.024
840	0.776	0.175	-0.026
841	0.836	0.145	-0.028
842	0.888	0.089	-0.03
843	0.934	0.037	-0.033
844	0.968	0.037	-0.038
845	0.138	0.254	-0.002
846	0.191	0.268	-0.004
847	0.247	0.244	-0.007
848	0.3	0.222	-0.009
849	0.365	0.261	-0.011
850	0.438	0.254	-0.013
851	0.494	0.229	-0.016
852	0.561	0.229	-0.018
853	0.634	0.222	-0.023
854	0.687	0.2	-0.024
855	0.75	0.204	-0.027
856	0.808	0.177	-0.028
857	0.879	0.099	-0.032
858	0.93	0.042	-0.036
859	0.959	0.01	-0.04
860	0.154	0.421	-0.004
861	0.2	0.406	-0.006
862	0.274	0.399	-0.009
863	0.323	0.382	-0.012
864	0.394	0.377	-0.013
865	0.469	0.33	-0.015
866	0.532	0.298	-0.016
867	0.605	0.328	-0.018
868	0.663	0.3	-0.02
869	0.721	0.273	-0.022
870	0.788	0.2	-0.026
871	0.839	0.143	-0.029
872	0.899	0.076	-0.035
873	0.95	0.02	-0.041
874	0.966	0.002	-0.047
875	0.131	0.52	-0.004
876	0.196	0.485	-0.007
877	0.258	0.416	-0.012
878	0.331	0.409	-0.01012
879	0.405	0.401	-0.01644
880	0.463	0.411	-0.02238
881	0.545	0.32	-0.0322
882	0.612	0.32	-0.04445
883	0.685	0.276	-0.05192
884	0.75	0.241	-0.05835
885	0.812	0.172	-0.06521
886	0.879	0.099	-0.07311

887	0.932	0.039	-0.0855
888	0.963	0.005	-0.08522
889	0.136	0.293	0.005513
890	0.187	0.31	0.000332
891	0.242	0.323	-0.00417
892	0.305	0.254	-0.0128
893	0.343	0.249	-0.01684
894	0.387	0.273	-0.01989
895	0.436	0.219	-0.02793
896	0.492	0.195	-0.0333
897	0.529	0.19	-0.03927
898	0.574	0.214	-0.03961
899	0.616	0.204	-0.04728
900	0.645	0.246	-0.04596
901	0.705	0.254	-0.05361
902	0.719	0.239	-0.05697
903	0.747	0.244	-0.05991
904	0.756	0.234	-0.06045
905	0.774	0.177	-0.06524
906	0.792	0.195	-0.062
907	0.808	0.177	-0.06797
908	0.85	0.131	-0.07568
909	0.89	0.086	-0.07701
910	0.888	0.089	-0.0768
911	0.908	0.067	-0.08194
912	0.908	0.067	-0.07915
913	0.919	0.054	-0.08077
914	0.903	0.071	-0.07834
915	0.921	0.052	-0.08192
916	0.917	0.057	-0.07781
917	0.116	0.722	0.022624
918	0.185	0.608	0.015221
919	0.249	0.611	0.008875
920	0.318	0.534	-0.00042
921	0.356	0.567	-0.00733
922	0.403	0.515	-0.01307
923	0.456	0.419	-0.0235
924	0.498	0.224	-0.0304
925	0.538	0.328	-0.03516
926	0.592	0.379	-0.03739
927	0.61	0.323	-0.04381
928	0.643	0.249	-0.04927
929	0.672	0.18	-0.04967
930	0.699	0.224	-0.05431
931	0.705	0.254	-0.05183
932	0.725	0.195	-0.05835
933	0.752	0.165	-0.05884
934	0.781	0.17	-0.06632
935	0.785	0.128	-0.06551
936	0.812	0.135	-0.06929
937	0.843	0.138	-0.06867
938	0.852	0.128	-0.0751
939	0.843	0.138	-0.0726
940	0.848	0.133	-0.07005
941	0.865	0.113	-0.07485
942	0.854	0.126	-0.07578
943	0.879	0.099	-0.0735
944	0.897	0.079	-0.07624
945	0.12	0.458	0.014778
946	0.178	0.394	0.00657
947	0.209	0.36	0.000536
948	0.26	0.34	-0.00354
949	0.276	0.286	-0.0062
950	0.307	0.325	-0.00939
951	0.352	0.202	-0.01832
952	0.389	0.271	-0.0203
953	0.465	0.224	-0.0323
954	0.538	0.143	-0.04127
955	0.556	0.234	-0.03903
956	0.603	0.219	-0.04255
957	0.621	0.163	-0.04791
958	0.652	0.165	-0.05175
959	0.667	0.185	-0.05271
960	0.705	0.18	-0.05671
961	0.723	0.16	-0.06079
962	0.772	0.143	-0.06545
963	0.808	0.14	-0.06492
964	0.808	0.14	-0.07113
965	0.85	0.131	-0.07568
966	0.868	0.111	-0.07056
967	0.874	0.103	-0.07682

968	0.892	0.084	-0.07818
969	0.885	0.091	-0.07359
970	0.901	0.074	-0.07802
971	0.883	0.094	-0.07938
972	0.899	0.076	-0.08268
973	0.138	0.254	0.003758
974	0.189	0.234	-0.00243
975	0.258	0.342	-0.00566
976	0.305	0.328	-0.00945
977	0.338	0.254	-0.01479
978	0.385	0.239	-0.02248
979	0.429	0.19	-0.02935
980	0.481	0.244	-0.03226
981	0.514	0.17	-0.03899
982	0.554	0.2	-0.0404
983	0.57	0.256	-0.03807
984	0.596	0.227	-0.04036
985	0.623	0.234	-0.04479
986	0.647	0.207	-0.0471
987	0.683	0.131	-0.05682
988	0.712	0.135	-0.05767
989	0.741	0.14	-0.06402
990	0.792	0.121	-0.06362
991	0.796	0.116	-0.06572
992	0.845	0.099	-0.07478
993	0.857	0.123	-0.0696
994	0.872	0.106	-0.0729
995	0.883	0.094	-0.07329
996	0.892	0.084	-0.07631
997	0.912	0.062	-0.07962
998	0.899	0.076	-0.08284
999	0.919	0.054	-0.08288
1000	0.91	0.064	-0.08129
1001	0.127	0.303	0.008583
1002	0.189	0.308	0.002722
1003	0.271	0.365	-0.00369
1004	0.34	0.288	-0.01356
1005	0.383	0.278	-0.01814
1006	0.407	0.325	-0.021
1007	0.474	0.251	-0.02911
1008	0.458	0.232	-0.02784
1009	0.501	0.296	-0.03124
1010	0.536	0.182	-0.0385
1011	0.581	0.207	-0.04141
1012	0.623	0.197	-0.04911
1013	0.659	0.268	-0.04718
1014	0.681	0.207	-0.05087
1015	0.701	0.222	-0.05067
1016	0.723	0.234	-0.05181
1017	0.743	0.212	-0.05559
1018	0.772	0.18	-0.06545
1019	0.805	0.143	-0.0681
1020	0.825	0.084	-0.06784
1021	0.859	0.121	-0.07625
1022	0.885	0.091	-0.07466
1023	0.872	0.106	-0.07789
1024	0.861	0.118	-0.0751
1025	0.865	0.113	-0.0753
1026	0.877	0.064	-0.0762
1027	0.87	0.108	-0.07566
1028	0.877	0.101	-0.07731
1029	0.116	0.389	0.012854
1030	0.158	0.342	0.004512
1031	0.218	0.387	0.001195
1032	0.28	0.355	-0.00621
1033	0.338	0.291	-0.0158
1034	0.38	0.281	-0.01744
1035	0.427	0.266	-0.02481
1036	0.492	0.268	-0.03276
1037	0.543	0.175	-0.03855
1038	0.561	0.192	-0.03894
1039	0.576	0.212	-0.04285
1040	0.583	0.241	-0.04221
1041	0.598	0.261	-0.04181
1042	0.643	0.286	-0.04393
1043	0.694	0.229	-0.05269
1044	0.701	0.222	-0.05134
1045	0.743	0.212	-0.06015
1046	0.776	0.212	-0.05922
1047	0.792	0.195	-0.0634
1048	0.81	0.175	-0.06241

1049	0.843	0.138	-0.07049
1050	0.859	0.121	-0.07011
1051	0.863	0.116	-0.07401
1052	0.868	0.111	-0.0749
1053	0.879	0.099	-0.07536
1054	0.885	0.091	-0.07545
1055	0.892	0.084	-0.08183
1056	0.877	0.101	-0.07782
1057	0.109	0.323	0.010133
1058	0.194	0.266	-0.00131
1059	0.24	0.251	-0.00546
1060	0.289	0.123	-0.01738
1061	0.336	0.182	-0.02003
1062	0.398	0.187	-0.02591
1063	0.449	0.131	-0.02986
1064	0.485	0.165	-0.03359
1065	0.532	0.187	-0.03932
1066	0.567	0.148	-0.04022
1067	0.61	0.175	-0.04731
1068	0.636	0.145	-0.04887
1069	0.652	0.165	-0.04816
1070	0.685	0.165	-0.05629
1071	0.719	0.165	-0.06084
1072	0.739	0.18	-0.06092
1073	0.794	0.118	-0.0695
1074	0.841	0.103	-0.0725
1075	0.852	0.128	-0.07004
1076	0.874	0.103	-0.07605
1077	0.883	0.094	-0.07348
1078	0.874	0.103	-0.07801
1079	0.874	0.103	-0.07585
1080	0.87	0.108	-0.07685
1081	0.917	0.057	-0.07798
1082	0.908	0.067	-0.08083
1083	0.899	0.076	-0.07691
1084	0.883	0.094	-0.07301
1085	0.156	0.419	0.008478
1086	0.185	0.424	0.006884
1087	0.24	0.362	-0.00176
1088	0.271	0.18	-0.01136
1089	0.331	0.187	-0.01752
1090	0.389	0.197	-0.02263
1091	0.429	0.227	-0.02711
1092	0.474	0.214	-0.03067
1093	0.501	0.148	-0.03792
1094	0.552	0.165	-0.03845
1095	0.587	0.2	-0.04605
1096	0.625	0.158	-0.05084
1097	0.665	0.187	-0.05187
1098	0.696	0.19	-0.05204
1099	0.71	0.175	-0.05706
1100	0.727	0.192	-0.05886
1101	0.765	0.15	-0.06066
1102	0.801	0.185	-0.06747
1103	0.83	0.116	-0.0732
1104	0.839	0.143	-0.06922
1105	0.85	0.131	-0.06973
1106	0.854	0.126	-0.07128
1107	0.888	0.089	-0.07733
1108	0.894	0.081	-0.07621
1109	0.901	0.074	-0.07921
1110	0.883	0.094	-0.07329
1111	0.894	0.081	-0.081
1112	0.892	0.084	-0.07988
1113	0.122	0.345	0.007509
1114	0.194	0.303	-0.0006
1115	0.265	0.298	-0.00661
1116	0.34	0.251	-0.01705
1117	0.4	0.222	-0.0225
1118	0.456	0.271	-0.02612
1119	0.476	0.249	-0.03037
1120	0.503	0.219	-0.03188
1121	0.541	0.251	-0.03448
1122	0.574	0.251	-0.03947
1123	0.634	0.185	-0.04655
1124	0.67	0.219	-0.05358
1125	0.707	0.214	-0.05485
1126	0.745	0.172	-0.05979
1127	0.761	0.155	-0.06266
1128	0.79	0.16	-0.06634
1129	0.803	0.145	-0.0692

1130	0.821	0.126	-0.07247
1131	0.821	0.163	-0.06906
1132	0.861	0.118	-0.07682
1133	0.868	0.111	-0.07828
1134	0.89	0.086	-0.07578
1135	0.888	0.089	-0.07884
1136	0.885	0.091	-0.07607
1137	0.894	0.081	-0.07645
1138	0.897	0.079	-0.0787
1139	0.894	0.081	-0.08214
1140	0.91	0.064	-0.07752
1141	0.113	0.355	0.011368
1142	0.202	0.367	0.001251
1143	0.265	0.261	-0.00938
1144	0.303	0.219	-0.0141
1145	0.374	0.251	-0.02073
1146	0.405	0.18	-0.02602
1147	0.449	0.241	-0.02813
1148	0.469	0.293	-0.02865
1149	0.512	0.246	-0.03232
1150	0.541	0.251	-0.03747
1151	0.607	0.251	-0.04241
1152	0.63	0.264	-0.0455
1153	0.667	0.222	-0.05204
1154	0.687	0.273	-0.05005
1155	0.732	0.187	-0.05753
1156	0.743	0.249	-0.05659
1157	0.736	0.219	-0.05537
1158	0.754	0.236	-0.06074
1159	0.761	0.229	-0.05742
1160	0.783	0.204	-0.06057
1161	0.805	0.18	-0.06629
1162	0.823	0.123	-0.07246
1163	0.848	0.133	-0.06822
1164	0.854	0.126	-0.07223
1165	0.854	0.126	-0.07284
1166	0.861	0.081	-0.07462
1167	0.885	0.091	-0.07555
1168	0.894	0.081	-0.07743
1169	0.127	0.266	0.0049
1170	0.185	0.239	-0.00092
1171	0.209	0.212	-0.00569
1172	0.254	0.2	-0.00904
1173	0.3	0.222	-0.01349
1174	0.358	0.268	-0.01574
1175	0.432	0.224	-0.02755
1176	0.474	0.177	-0.03037
1177	0.538	0.217	-0.03659
1178	0.581	0.244	-0.0416
1179	0.607	0.288	-0.04303
1180	0.641	0.288	-0.04394
1181	0.674	0.251	-0.04751
1182	0.705	0.254	-0.05124
1183	0.747	0.207	-0.05991
1184	0.799	0.187	-0.06522
1185	0.812	0.172	-0.06649
1186	0.843	0.138	-0.07457
1187	0.881	0.096	-0.07959
1188	0.883	0.094	-0.07566
1189	0.883	0.094	-0.08045
1190	0.894	0.081	-0.0745
1191	0.894	0.081	-0.07645
1192	0.91	0.064	-0.08213
1193	0.91	0.064	-0.08063
1194	0.914	0.059	-0.08213
1195	0.91	0.064	-0.08258
1196	0.908	0.067	-0.0813
1197	0.145	0.431	0.00902
1198	0.22	0.458	0.001828
1199	0.265	0.483	0.000726
1200	0.316	0.463	-0.00399
1201	0.367	0.48	-0.0108
1202	0.416	0.315	-0.01871
1203	0.465	0.261	-0.02893
1204	0.518	0.202	-0.03655
1205	0.547	0.281	-0.03468
1206	0.59	0.234	-0.03881
1207	0.59	0.308	-0.0405
1208	0.612	0.246	-0.0452
1209	0.627	0.192	-0.04929
1210	0.652	0.202	-0.05148

1211	0.703	0.145	-0.05946
1212	0.734	0.148	-0.06154
1213	0.779	0.209	-0.05842
1214	0.814	0.17	-0.07071
1215	0.85	0.131	-0.07503
1216	0.859	0.121	-0.07171
1217	0.881	0.096	-0.07969
1218	0.881	0.096	-0.07812
1219	0.899	0.076	-0.08223
1220	0.919	0.054	-0.07812
1221	0.912	0.062	-0.08261
1222	0.93	0.042	-0.08167
1223	0.928	0.044	-0.08207
1224	0.921	0.052	-0.08494
1225	0.129	0.337	0.007519
1226	0.171	0.291	0.003173
1227	0.238	0.291	-0.00345
1228	0.287	0.273	-0.01038
1229	0.323	0.234	-0.01638
1230	0.358	0.232	-0.02065
1231	0.427	0.229	-0.02643
1232	0.492	0.268	-0.02981
1233	0.538	0.291	-0.03221
1234	0.565	0.224	-0.03795
1235	0.623	0.197	-0.0495
1236	0.643	0.212	-0.04932
1237	0.67	0.219	-0.05068
1238	0.687	0.2	-0.05181
1239	0.745	0.209	-0.05611
1240	0.756	0.234	-0.06004
1241	0.774	0.177	-0.06155
1242	0.785	0.165	-0.0625
1243	0.812	0.172	-0.06598
1244	0.828	0.155	-0.07155
1245	0.848	0.133	-0.07461
1246	0.874	0.103	-0.07898
1247	0.899	0.076	-0.08089
1248	0.905	0.069	-0.08259
1249	0.901	0.074	-0.07531
1250	0.908	0.067	-0.08278
1251	0.901	0.074	-0.08289
1252	0.905	0.069	-0.08058
1253	0.122	0.382	0.009064
1254	0.198	0.335	0.001478
1255	0.231	0.335	-0.00168
1256	0.276	0.323	-0.00608
1257	0.323	0.271	-0.01163
1258	0.336	0.256	-0.01587
1259	0.365	0.261	-0.01717
1260	0.392	0.305	-0.02061
1261	0.443	0.286	-0.02528
1262	0.487	0.273	-0.03282
1263	0.527	0.229	-0.03389
1264	0.578	0.172	-0.04258
1265	0.618	0.202	-0.0453
1266	0.645	0.172	-0.05289
1267	0.665	0.187	-0.04873
1268	0.681	0.207	-0.05469
1269	0.725	0.158	-0.0597
1270	0.754	0.163	-0.06282
1271	0.776	0.175	-0.062
1272	0.814	0.133	-0.06659
1273	0.848	0.133	-0.07116
1274	0.848	0.133	-0.07554
1275	0.877	0.101	-0.07586
1276	0.897	0.079	-0.07846
1277	0.903	0.071	-0.07564
1278	0.892	0.084	-0.07826
1279	0.923	0.049	-0.08424
1280	0.921	0.052	-0.07828
1281	0.12	0.384	0.012608
1282	0.162	0.3	0.005048
1283	0.218	0.313	-0.0034
1284	0.267	0.296	-0.00659
1285	0.294	0.229	-0.01149
1286	0.336	0.33	-0.01273
1287	0.374	0.362	-0.01567
1288	0.425	0.268	-0.02501
1289	0.458	0.268	-0.02517
1290	0.485	0.239	-0.03262
1291	0.525	0.195	-0.03651

1292	0.585	0.202	-0.04163
1293	0.605	0.291	-0.03965
1294	0.612	0.209	-0.04833
1295	0.623	0.234	-0.04455
1296	0.665	0.224	-0.05044
1297	0.707	0.251	-0.05271
1298	0.736	0.219	-0.05463
1299	0.788	0.163	-0.06556
1300	0.805	0.18	-0.06459
1301	0.83	0.116	-0.07024
1302	0.845	0.135	-0.06772
1303	0.859	0.084	-0.0784
1304	0.861	0.118	-0.07498
1305	0.885	0.091	-0.08022
1306	0.901	0.074	-0.07936
1307	0.912	0.062	-0.08437
1308	0.912	0.062	-0.08327
1309	0.118	0.387	0.011713
1310	0.189	0.345	0.002399
1311	0.245	0.394	-0.00187
1312	0.278	0.283	-0.00931
1313	0.343	0.286	-0.01326
1314	0.4	0.333	-0.01641
1315	0.438	0.291	-0.02386
1316	0.483	0.352	-0.02697
1317	0.536	0.33	-0.03372
1318	0.574	0.288	-0.03989
1319	0.63	0.264	-0.0455
1320	0.692	0.268	-0.05075
1321	0.705	0.254	-0.05285
1322	0.75	0.204	-0.057
1323	0.768	0.148	-0.0604
1324	0.81	0.175	-0.06931
1325	0.836	0.108	-0.07462
1326	0.863	0.116	-0.07268
1327	0.883	0.094	-0.07919
1328	0.901	0.074	-0.08004
1329	0.894	0.081	-0.07946
1330	0.91	0.064	-0.07953
1331	0.917	0.057	-0.07856
1332	0.912	0.062	-0.08468
1333	0.905	0.069	-0.07897
1334	0.903	0.071	-0.07884
1335	0.899	0.076	-0.07976
1336	0.885	0.091	-0.07695
1337	0.149	0.241	0.002017
1338	0.211	0.209	-0.00475
1339	0.291	0.195	-0.01214
1340	0.349	0.204	-0.02025
1341	0.394	0.192	-0.02391
1342	0.44	0.177	-0.02742
1343	0.478	0.172	-0.03275
1344	0.503	0.182	-0.03571
1345	0.554	0.236	-0.04065
1346	0.581	0.207	-0.0436
1347	0.614	0.207	-0.04349
1348	0.641	0.214	-0.04968
1349	0.683	0.167	-0.05329
1350	0.712	0.209	-0.05375
1351	0.734	0.185	-0.05965
1352	0.763	0.153	-0.06142
1353	0.788	0.163	-0.0651
1354	0.816	0.167	-0.06414
1355	0.814	0.133	-0.06698
1356	0.821	0.163	-0.06462
1357	0.848	0.133	-0.07363
1358	0.852	0.128	-0.06854
1359	0.877	0.101	-0.07917
1360	0.892	0.084	-0.07622
1361	0.914	0.059	-0.0839
1362	0.905	0.069	-0.07884
1363	0.894	0.081	-0.08092
1364	0.917	0.057	-0.0797
1365	0.12	0.347	0.008492
1366	0.18	0.318	0.003356
1367	0.247	0.281	-0.00645
1368	0.294	0.303	-0.01021
1369	0.365	0.224	-0.01918
1370	0.429	0.19	-0.02673
1371	0.483	0.204	-0.03461
1372	0.521	0.236	-0.03665

1373	0.547	0.244	-0.03603
1374	0.592	0.305	-0.0399
1375	0.614	0.207	-0.04738
1376	0.641	0.177	-0.05105
1377	0.656	0.234	-0.04688
1378	0.667	0.222	-0.04826
1379	0.723	0.197	-0.0555
1380	0.745	0.209	-0.05579
1381	0.785	0.202	-0.06105
1382	0.79	0.197	-0.05957
1383	0.854	0.126	-0.07517
1384	0.877	0.101	-0.07441
1385	0.863	0.079	-0.076
1386	0.863	0.116	-0.07648
1387	0.883	0.094	-0.08007
1388	0.89	0.086	-0.07621
1389	0.917	0.057	-0.0777
1390	0.921	0.052	-0.07823
1391	0.917	0.057	-0.07873
1392	0.914	0.059	-0.08184
1393	0.122	0.419	0.011897
1394	0.165	0.409	0.008929
1395	0.236	0.293	-0.00364
1396	0.291	0.232	-0.01095
1397	0.347	0.244	-0.01531
1398	0.389	0.197	-0.02245
1399	0.44	0.214	-0.03006
1400	0.503	0.219	-0.03269
1401	0.545	0.246	-0.03666
1402	0.592	0.195	-0.04615
1403	0.627	0.229	-0.0428
1404	0.683	0.241	-0.04913
1405	0.725	0.195	-0.05921
1406	0.761	0.192	-0.06004
1407	0.79	0.16	-0.06792
1408	0.812	0.135	-0.06996
1409	0.839	0.106	-0.07533
1410	0.861	0.118	-0.07448
1411	0.859	0.121	-0.07624
1412	0.877	0.101	-0.0787
1413	0.901	0.074	-0.08072
1414	0.901	0.074	-0.08094
1415	0.923	0.049	-0.07972
1416	0.928	0.044	-0.08498
1417	0.912	0.062	-0.08418
1418	0.919	0.054	-0.0825
1419	0.921	0.052	-0.08315
1420	0.912	0.062	-0.07823
1421	0.116	0.463	0.013879
1422	0.154	0.384	0.00727
1423	0.191	0.342	0.003455
1424	0.234	0.443	0.000941
1425	0.291	0.453	-0.00372
1426	0.343	0.36	-0.01403
1427	0.36	0.229	-0.01822
1428	0.4	0.222	-0.02521
1429	0.438	0.217	-0.02952
1430	0.469	0.219	-0.03017
1431	0.496	0.153	-0.03529
1432	0.543	0.249	-0.03446
1433	0.565	0.187	-0.04083
1434	0.587	0.236	-0.04138
1435	0.625	0.232	-0.04467
1436	0.676	0.212	-0.04956
1437	0.707	0.177	-0.05262
1438	0.739	0.18	-0.05964
1439	0.781	0.17	-0.06649
1440	0.819	0.128	-0.07244
1441	0.845	0.099	-0.07582
1442	0.863	0.116	-0.07228
1443	0.87	0.071	-0.07696
1444	0.912	0.062	-0.07914
1445	0.912	0.062	-0.0772
1446	0.901	0.074	-0.07944
1447	0.89	0.086	-0.08049
1448	0.901	0.074	-0.08041
1449	0.142	0.286	0.004524
1450	0.189	0.271	0.0004
1451	0.245	0.283	-0.00531
1452	0.314	0.281	-0.01366
1453	0.363	0.227	-0.01836

1454	0.405	0.254	-0.02102
1455	0.454	0.273	-0.02575
1456	0.474	0.214	-0.03286
1457	0.525	0.195	-0.03978
1458	0.572	0.18	-0.04411
1459	0.61	0.249	-0.04196
1460	0.647	0.207	-0.04865
1461	0.685	0.239	-0.05022
1462	0.727	0.155	-0.06172
1463	0.73	0.153	-0.06211
1464	0.743	0.212	-0.06016
1465	0.761	0.192	-0.0608
1466	0.803	0.182	-0.06396
1467	0.796	0.19	-0.06635
1468	0.83	0.153	-0.07183
1469	0.839	0.106	-0.0725
1470	0.85	0.131	-0.06987
1471	0.872	0.106	-0.07768
1472	0.879	0.099	-0.07819
1473	0.901	0.074	-0.08116
1474	0.901	0.074	-0.07568
1475	0.914	0.059	-0.07921
1476	0.921	0.052	-0.08596
1477	0.111	0.246	0.006653
1478	0.149	0.241	0.00313
1479	0.2	0.259	-0.00124
1480	0.258	0.232	-0.00906
1481	0.298	0.187	-0.01376
1482	0.349	0.241	-0.01847
1483	0.389	0.234	-0.02162
1484	0.461	0.266	-0.02773
1485	0.503	0.256	-0.03307
1486	0.532	0.261	-0.03449
1487	0.581	0.244	-0.04098
1488	0.616	0.241	-0.04262
1489	0.659	0.158	-0.05353
1490	0.674	0.177	-0.0537
1491	0.701	0.185	-0.05736
1492	0.721	0.236	-0.05539
1493	0.747	0.244	-0.05494
1494	0.763	0.227	-0.05685
1495	0.79	0.197	-0.06431
1496	0.816	0.167	-0.06449
1497	0.832	0.076	-0.07156
1498	0.85	0.131	-0.07261
1499	0.843	0.138	-0.074
1500	0.863	0.116	-0.07182
1501	0.883	0.094	-0.07594
1502	0.905	0.069	-0.07779
1503	0.91	0.064	-0.07888
1504	0.917	0.057	-0.08503
1505	0.136	0.367	0.009352
1506	0.178	0.246	-0.00016
1507	0.225	0.232	-0.00545
1508	0.263	0.227	-0.00896
1509	0.325	0.232	-0.01652
1510	0.394	0.229	-0.02037
1511	0.445	0.209	-0.02722
1512	0.496	0.19	-0.0353
1513	0.552	0.165	-0.04198
1514	0.592	0.158	-0.04729
1515	0.63	0.227	-0.0434
1516	0.681	0.17	-0.05245
1517	0.719	0.202	-0.05591
1518	0.774	0.177	-0.06517
1519	0.805	0.18	-0.06647
1520	0.812	0.172	-0.0644
1521	0.801	0.148	-0.06888
1522	0.841	0.14	-0.07022
1523	0.861	0.118	-0.07655
1524	0.89	0.086	-0.07587
1525	0.89	0.086	-0.0771
1526	0.91	0.064	-0.07661
1527	0.894	0.081	-0.07475
1528	0.879	0.099	-0.0733
1529	0.872	0.106	-0.0729
1530	0.879	0.099	-0.07975
1531	0.877	0.101	-0.07849
1532	0.897	0.079	-0.07535
1533	0.76	0.207	-0.05647
1534	0.796	0.197	-0.06074
1535	0.822	0.086	-0.07413
1536	0.83	0.161	-0.06987
1537	0.833	0.158	-0.06664
1538	0.833	0.136	-0.07
1539	0.863	0.124	-0.07359
1540	0.875	0.079	-0.07324
1541	0.89	0.074	-0.08167
1542	0.887	0.067	-0.07605
1543	0.863	0.124	-0.07716
1544	0.88	0.106	-0.07629
1545	0.897	0.067	-0.0757
1546	0.911	0.082	-0.08255
1547	0.907	0.077	-0.08234
1548	0.884	0.069	-0.07785
1549	0.112	0.429	0.013326
1550	0.155	0.419	0.010362
1551	0.226	0.313	-0.00253
1552	0.271	0.242	-0.00815
1553	0.337	0.264	-0.01428
1554	0.379	0.227	-0.01999
1555	0.43	0.234	-0.02474
1556	0.473	0.229	-0.0327
1557	0.515	0.256	-0.03429
1558	0.562	0.225	-0.03628
1559	0.597	0.259	-0.04383
1560	0.122	0.296	0.008772
1561	0.19	0.289	-0.0002
1562	0.215	0.276	-0.00268
1563	0.295	0.278	-0.01207
1564	0.37	0.264	-0.01907
1565	0.428	0.242	-0.02676
1566	0.502	0.266	-0.03135
1567	0.578	0.244	-0.04357
1568	0.633	0.237	-0.04519
1569	0.735	0.254	-0.05237
1570	0.786	0.197	-0.06174
1571	0.854	0.113	-0.07618
1572	0.901	0.062	-0.07981
1573	0.942	0.047	-0.08149
1574	0.13	0.372	0.008616
1575	0.184	0.36	0.004184
1576	0.204	0.389	0.00353
1577	0.291	0.367	-0.00526
1578	0.367	0.33	-0.0162
1579	0.571	0.195	-0.04168
1580	0.622	0.18	-0.04708
1581	0.637	0.205	-0.04918
1582	0.707	0.202	-0.05442
1583	0.732	0.148	-0.06075
1584	0.782	0.182	-0.06527
1585	0.806	0.141	-0.06896
1586	0.84	0.114	-0.07239
1587	0.845	0.123	-0.07339
1588	0.875	0.101	-0.07304
1589	0.873	0.124	-0.07048
1590	0.879	0.106	-0.0783
1591	0.888	0.077	-0.0744
1592	0.873	0.101	-0.07838
1593	0.864	0.091	-0.07803
1594	0.869	0.096	-0.07313
1595	0.869	0.106	-0.07695
1596	0.869	0.106	-0.07422
